# Heterogeneously Expressed GadAB Identified by Single-Cell RNA Sequencing Mediates Antibiotic Tolerance Under Acid Stress

**DOI:** 10.3390/microorganisms14102180

**Published:** 2026-09-29

**Authors:** Hebin Liao, Bingxue Ning, Xinyi Zheng, Zixuan Zhang, Guoyang He, Manqi Xiao, Kangxinhe Zhang, Aimin Jia, Xiaodan Yan

**Affiliations:** 1 School of Laboratory Medicine & Translational Medicine Research Center, North Sichuan Medical College, Nanchong 637000, China; liaohebin@nsmc.edu.cn (H.L.);; 2 Institute of Infection and Immunity, Henan Academy of Innovation in Medical Science, Zhengzhou 450000, China

**Keywords:** heterogeneity, single-cell RNA sequencing, GadA, GadB, acid stress, antibiotic tolerance

## Abstract

Bacterial population heterogeneity serves as the intrinsic basis for persister formation. Single-cell RNA sequencing is a powerful tool for investigating such heterogeneity. In this study, we analyzed single-cell transcriptomic data, previously generated via RiboD-PETRI-seq, from exponential-phase *Escherichia coli* MG1655 populations. We identified subpopulations with heterogeneous expression of *gadA* and *gadB*. Dual-fluorescent reporter strains were subsequently utilized to verify the heterogeneous protein abundance of GadA and GadB across the bacterial community. The expression levels of these two proteins were positively correlated within individual cells. We further observed that the naturally occurring GadA/GadB-low subpopulation exhibited a higher persister ratio than the GadA/GadB-high subpopulation under acid stress, and a similar phenotype was recapitulated in the Δ*gadAB* mutant. Integrated transcriptomic and metabolomic analyses collectively demonstrated that the *gadAB* double mutant exhibits a dormant-like state upon acid exposure. This study confirms heterogeneous *gadA* and *gadB* expression within bacterial populations. The GadA/GadB-low subpopulation exhibits enhanced antibiotic tolerance under acid stress. This work offers theoretical insights into the acid and antibiotic tolerance mechanisms of enteric pathogens in the acidic gastrointestinal niche.

## 1. Introduction

Persister cells constitute a specialized phenotypic subpopulation within bacterial populations that can tolerate lethal concentrations of antibiotics. After antibiotics are removed, persister cells can resuscitate [1]. They are a major contributor to chronic and recurrent infections, including urinary tract infections, tuberculosis, cystic fibrosis–associated pulmonary infections, and device-associated biofilm infections [1,2,3,4,5,6,7]. Therefore, elucidating the mechanisms underlying persister formation is of clinical importance.

Persister formation is inherently a subpopulation phenomenon: even in clonal cultures, only a minor fraction of cells enters the persistent state, typically on the order of 0.0001–0.01% in exponential phase and below 1% in stationary or death phase [8,9]. This heterogeneity arises primarily from cell-to-cell variation in gene expression [10]. Accordingly, studying genes with heterogeneous expression will help us understand the molecular mechanisms of persister formation. Single-cell RNA sequencing is a powerful tool for identifying heterogeneously expressed genes that may underlie persister formation. We previously developed the RiboD-PETRI-seq technique and applied it to reveal transcriptional heterogeneity in *E. coli* biofilm subpopulations [11,12].

The GadA/GadB proteins constitute a glutamate-dependent acid resistance system in Gram-negative bacteria [13,14,15,16,17]. The glutamate/γ-aminobutyrate antiporter GadC imports extracellular glutamate, which GadA and GadB decarboxylate to γ-aminobutyrate (GABA) in a proton-consuming reaction [18,19,20]. The resulting GABA is subsequently exported by GadC, collectively maintaining near-neutral intracellular pH under extreme acid stress [18,19,21]. Transcription of *gadA* and *gadB* is governed by a complex regulatory network involving GadE, GadX, GadW, RpoS, H-NS, CRP, and the two-component system EvgAS [15,22,23,24,25,26,27]. While *gadA*/*gadB* expression has been extensively characterized at the bulk population level, whether these genes exhibit heterogeneous expression at the single-cell level, whether GadA and GadB proteins exhibit coordinated heterogeneous expression at the single-cell level, and whether such heterogeneity has functional consequences for antibiotic tolerance under acid stress remains unknown.

Considering its vital function in acid defense [13,27] and the multi-layered regulation of *gadA*/*gadB*, we hypothesized that stochastic variation in upstream regulatory factors could generate heterogeneous GadA/GadB expression within a population, and that subpopulations with low GadA/GadB expression might exhibit altered persister formation under acid stress. In this study, we combined single-cell transcriptomics, dual-fluorescence reporter imaging, FACS-based functional assays, and integrated transcriptomic and metabolomic analyses to test this hypothesis.

These findings help explain how naturally occurring heterogeneity in the Gad acid-resistance system modulates persister formation under acidic conditions in vitro, providing a conceptual framework for future investigations into how enteric bacteria may survive antibiotic treatment in the acidic gastrointestinal niche.

## 2. Materials and Methods

### 2.1. Bacterial Strains and Growth Conditions

All the strains and plasmids used in this study are described in Table S1. Unless otherwise stated, all the strains were cultured in Luria broth (LB) medium (Sangon Biotech (Shanghai) Co., Ltd., Shanghai, China) under routine growth conditions at 37 °C and 200 rpm. LBK medium was prepared as follows: 10 g of tryptone (Oxoid Ltd., Basingstoke, UK), 5 g of yeast extract (Oxoid Ltd., Basingstoke, UK) and 7.45 g of KCl (Sangon Biotech (Shanghai) Co., Ltd., Shanghai, China) were dissolved in double-distilled water to a final volume of 1 L, followed by autoclave sterilization [17,28]. LBKG medium was prepared by supplementing LBK medium with 10 mM glutamate (Sangon Biotech (Shanghai) Co., Ltd., Shanghai, China), and the pH was adjusted to 4.4. Unless otherwise stated, LBKG medium in this study refers to this pH 4.4 formulation [17,28]. When necessary, the bacteria were cultured with kanamycin (Sangon Biotech (Shanghai) Co., Ltd., Shanghai, China) at a final concentration of 50 μg/mL and chloramphenicol (Sangon Biotech (Shanghai) Co., Ltd., Shanghai, China) at 20 μg/mL.

Single-cell transcriptomics and fluorescent-reporter experiments were performed in *E. coli* MG1655, whereas gene-deletion and complementation experiments were performed in *E. coli* BW25113 using strains from the Keio collection. Both strains are K-12 derivatives with a conserved *gadA*/*gadB* regulatory network.

### 2.2. Strain Construction

Genomic modification was performed via λ-Red-mediated homologous recombination [29]. To generate the double mutant BW25113 Δ*gadA*::*kanR* Δ*gadB*::*cat*, we deleted the *gadB* gene using BW25113 Δ*gadA*::*kanR* as the parental background strain. Briefly, three fragments, including the 500 bp upstream homologous arm of the *gadB* coding sequence, *cat* (chloramphenicol resistance cassette), and the 500 bp downstream homologous arm of the *gadB* coding region, were fused into a single DNA fragment. This fragment was subsequently electroporated into BW25113 Δ*gadA*::*kanR*, which carried the pSIM6 plasmid. For the preparation of electrocompetent cells, the strain harboring plasmid pSIM6 was cultured to mid-exponential phase in a total volume of 3 mL at 30 °C. The λ-Red recombinase genes encoded by pSIM6 were induced by heat shock at 37 °C for 20 min. The bacterial cells were then immediately washed twice with ice-cold 10% glycerol (Sangon Biotech (Shanghai) Co., Ltd., Shanghai, China) and finally resuspended in 100 μL of ice-cold 10% glycerol to yield electrocompetent cells. Transformants were screened on an LB plate supplemented with 20 μg/mL chloramphenicol, followed by PCR and DNA sequencing verification to confirm the replacement of the endogenous *gadB* gene with the *cat* gene. Verified-positive strains were cultured at 37 °C to cure the pSIM6 plasmid. The overnight bacterial culture was supplemented with 20% glycerol and stored at −80 °C. Bulk RNA-seq under acid stress confirmed that *gadC* expression was not reduced in the Δ*gadAB* mutant (instead, it was ~15-fold higher than in WT), indicating that the polar effect of the deletion cassette on downstream *gadC* does not confound the acid-stress phenotype. The minimum inhibitory concentration of ampicillin (Sangon Biotech (Shanghai) Co., Ltd., Shanghai, China) was also measured for the Δ*gadAB* mutant and was found to be identical to that of the parental strain (4 μg/mL for both).

To construct the GadB-GFP fluorescent reporter strain in MG1655, multiple fragments, including the 500 bp sequence upstream of the stop codon within the *gadB* coding region, the *gfp* gene, the *sacB* cassette, the *cat* gene, and the fragment covering the *gadB* stop codon plus its 500 bp downstream homologous arm, were fused into a single DNA construct. The fused fragment was subsequently electroporated into *E. coli* MG1655 carrying the pSIM6 plasmid. Transformants were selected on an LB plate supplemented with 20 μg/mL chloramphenicol, and positive clones were verified by PCR to confirm that the *gfp*-sacB cassette-*cat* was inserted upstream of the stop codon of the *gadB* coding sequence. Two fragments consisting of the 500 bp sequence upstream of the *gadB* stop codon, as well as the fragment containing the *gadB* stop codon and its 500 bp downstream homologous arm, were subsequently fused into a single DNA fragment. This fragment was subsequently electroporated into the aforementioned transformants, which were subsequently screened on LB plates supplemented with 10% sucrose (Sigma-Aldrich, St. Louis, MO, USA). Transformants were subjected to PCR and DNA sequencing verification to confirm the deletion of the *sacB* cassette *cat*. Positively verified strains were cultured at 37 °C to cure the pSIM6 plasmid. With the same strategy, we inserted the *mCherry* gene upstream of the stop codon of *gadA* in the MG1655 *gadB*-*gfp* background to generate the MG1655 *gadA*-*mcherry gadB*-*gfp* dual-fluorescent strain. Using the same genetic manipulation strategy, the *gfp* gene was inserted upstream of the stop codon of the *sppA* gene in the MG1655 background to construct the MG1655 *sppA*-*gfp* strain. The overnight bacterial culture was supplemented with 20% glycerol and stored at −80 °C.

To construct the *gadAB* expression plasmid, a seamless assembly strategy was employed. The pBAD-derived vector backbone, in which the original ampicillin resistance cassette had been substituted with a gentamicin resistance gene, was generated as the first PCR fragment. The second fragment consisted of the full-length *gadA* coding sequence together with the 210 bp region immediately upstream of the start codon, amplified from the genomic DNA of strain MG1655. The third fragment consisted of the full-length *gadB* coding sequence together with the 360 bp region immediately upstream of the start codon, amplified from the genomic DNA of strain MG1655. The three amplicons were joined in vitro via the 2× MultiF Seamless Assembly Mix (Cat. No. RK21020, ABclonal Technology, Wuhan, China). The assembly product was then introduced into competent *E. coli* DH5α cells by transformation. Transformants were selected on LB agar plates supplemented with 20 μg/mL gentamicin (Sangon Biotech (Shanghai) Co., Ltd., Shanghai, China), and correct assembly was confirmed by Sanger sequencing. The 210 bp and 360 bp upstream regions included in the *gadA* and *gadB* fragments contain their respective native promoters. Therefore, *gadA* and *gadB* are expressed from their native promoters rather than from the pBAD promoter. No L-arabinose was added in the complementation experiment.

### 2.3. Persister Counting Assay

Persister assays were performed on (i) cultures grown to the exponential, stationary, and death phases, (ii) acid-stressed cultures, and (iii) FACS-sorted GadA/GadB-high and -low subpopulations. For (i), strains were inoculated into LB medium, cultured overnight at 37 °C with shaking at 220 rpm, diluted 1:100 into fresh LB medium, and incubated for 3 h, 12 h, or 24 h to reach the exponential, stationary, and death phases, respectively. For (ii), strains were grown overnight in LBK medium, washed once with PBS, resuspended in LBKG at a 1:1 (*v*/*v*) ratio, diluted 1:10 into fresh LBKG medium, and incubated at 37 °C and 220 rpm for 2 h. For (iii), GadA/GadB-high and -low subpopulations sorted by FACS were exposed to acid stress in LBKG medium for 2 h. For all conditions, the pre-antibiotic viable count (CFU_0_) was determined immediately before antibiotic addition (i.e., after the 2-h acid stress period for conditions (ii) and (iii)), by serial dilution and plating onto LB agar. Parallel acid-only controls were performed, in which bacterial cultures were incubated in LBKG medium without antibiotics for identical durations, to assess cell death induced by acid stress alone. The cultures from (i) and (ii) were then diluted 1:20 into LB or LBKG medium, respectively, supplemented with 150 μg/mL ampicillin (Amp) and incubated at 37 °C with shaking at 220 rpm for 3 h. The sorted cells from (iii) were treated directly with 150 μg/mL Amp for 3 h or 3 μg/mL ciprofloxacin (Cip, Sangon Biotech (Shanghai) Co., Ltd., Shanghai, China) for 5 h. These treatment durations reached the second phase of biphasic bacterial killing, enabling reliable measurement of persister abundance (Figure S1). After treatment, cells were harvested by centrifugation (5000× *g*, 2 min, room temperature), resuspended in sterile PBS, serially diluted, and plated onto LB agar for CFU_t_ enumeration. Persister fractions were calculated as the ratio of CFU recovered after antibiotic treatment to those before antibiotic exposure (CFU_t_/CFU_0_) and expressed on a log_10_ scale. At least three biological replicates were performed for each experiment.

### 2.4. Microscopic Imaging and Fluorescence Analysis

The MG1655 *gadA*-*mcherry gadB*-*gfp* strain was inoculated into LB medium and cultured overnight at 37 °C with shaking at 220 rpm. Overnight cultures were diluted 1:100 into fresh liquid LB medium and incubated at 37 °C and 220 rpm for 3 h (exponential phase), 12 h (stationary phase), or 24 h (death phase). Bacterial pellets were washed once with PBS and deposited onto low-melting-point agarose blocks prepared in PBS for fluorescence imaging with a Nikon TI2 CTRE microscope (Nikon, Tokyo, Japan). mCherry fluorescence was captured with a TRITC filter cube (EX540/25, DM565, BA605/55). GFP fluorescence was captured with a FITC filter cube (EX465-495, DM505, BA512/55). Image analysis was performed with ImageJ software (Fiji, version 1.54f). For each individual cell, the mean fluorescence intensity was first corrected by subtracting the background fluorescence, and then normalized to the maximum signal within each dataset, with the maximum value set to 1000. At least three biological replicates were performed for each growth phase, and the results were consistent across replicates. To avoid batch effects introduced by variations in imaging conditions across independent sessions, the fluorescence distributions and correlation analyses shown in the results section are from one representative experiment. The consistency of results across biological replicates was verified.

### 2.5. Single-Cell RNA-Seq Data Analysis

The results of single-cell sequencing of *E. coli* MG1655 were downloaded from the GEO database (GSE337836). Data analysis was performed as described in our previous study [11]. After the matrix files were generated, single-cell data analysis was conducted using Seurat. Two separate Seurat objects were created using the CreateSeuratObject() function from the Seurat package (version 5.4.0), with the transposed count matrices as input. Genes detected in fewer than 5 cells were filtered out (min.cells = 5; a gene-level filter). For cell-level quality control, we applied min.features thresholds per replicate to balance cell retention while removing low-quality cells: 30 genes for Replicate 1 and 20 genes for Replicate 2, due to slight differences in sequencing depth. The upper limit was 4000 genes for both replicates. These thresholds yielded comparable numbers of high-quality cells between replicates (Replicate 1: 10,775 cells; Replicate 2: 11,913 cells). The percentage of mitochondrial genes was not examined because the data originated from prokaryotic (*E. coli*) transcripts. After filtering, each dataset was normalized using NormalizeData() (scale factor = 10,000), and variable features were identified using the vst method (FindVariableFeatures(), nfeatures = 500). The two preprocessed Seurat objects were integrated using the standard Seurat integration workflow. Integration anchors were identified with FindIntegrationAnchors() using canonical correlation analysis (CCA; dims = 1:20), followed by data integration using IntegrateData() (dims = 1:20). The resulting integrated object was then scaled, and principal component analysis (PCA) was performed (npcs = 30). The number of principal components (PCs) used for downstream analysis was determined by examining both the Elbow plot and the JackStraw procedure. These analyses indicated that the first 5 PCs captured the majority of the biologically relevant variance, with additional PCs contributing only marginal information and showing no distinct structure in the Uniform Manifold Approximation and Projection (UMAP) embeddings. Therefore, the first 5 PCs of the CCA-integrated PCA space were used for downstream analyses. UMAP was performed on the basis of these 5 PCs. A shared nearest neighbor graph was constructed, and cells were clustered using the Louvain algorithm with a resolution of 0.17. To evaluate the robustness of our clustering results to the analytical strategy, we performed three complementary sensitivity analyses. First, we tested alternative clustering resolutions (0.1, 0.25, and 0.5) and PC numbers (7 and 10) and different integration methods; all settings consistently identified the *gadA*/*gadB*-high Cluster (Figures S3 and S4). Second, we applied Harmony (version 2.0.5) to the CCA-integrated PCA space using RunHarmony() with group.by.vars = “orig.ident” [30], as a conservative correction for sequencing-run-specific technical noise, and repeated the downstream UMAP and clustering steps on the Harmony-corrected embeddings. The resulting clustering structure was highly similar to that obtained with CCA integration alone, and the *gadA*/*gadB*-high Cluster was reproducibly identified. Third, to assess whether the identified cell populations were reproducible within each sample independently, we performed clustering on each sample separately without cross-sample integration. Each sample was processed using the same preprocessing, dimensionality reduction, and clustering parameters as described above for the integrated analysis. No batch correction or integration across samples was applied in these analyses. The *gadA*/*gadB*-high Cluster was recovered in both replicates independently, confirming that this population is not an artifact of integration. Because the CCA-only and CCA with Harmony workflows yielded highly similar clustering structures, the CCA-integrated results are presented in Figure 1, Figures S3 and S5, and the Harmony-based comparison is provided in Figure S4. Clusters were visualized on the UMAP embedding using DimPlot(). Marker genes for each cluster were identified using FindAllMarkers(). The Wilcoxon rank sum test was used as the default test. Differentially expressed genes with a *p-adj* value less than 0.05 and a fold change greater than 2 were considered statistically significant for downstream analysis. The top five marker genes per cluster (based on average log2FC) were visualized using a heatmap (DoHeatmap()). Clusters were also annotated by known marker genes (e.g., *gadA* and *gadB*) using Seurat functions FeaturePlot() and VlnPlot().

### 2.6. Untargeted Microbial Metabolomics Assays and RNA-Sequencing

BW25113 WT and Δ*gadAB* strains were inoculated into LBK medium and cultured overnight at 37 °C with shaking at 220 rpm. The overnight cultures were washed once with PBS and resuspended in an equal volume of LBKG medium, followed by a 1:10 dilution into fresh LBKG medium. The cultures were incubated at 37 °C and 220 rpm for 2 h. The bacterial pellets were harvested, washed with PBS, and immediately frozen for subsequent analysis.

#### 2.6.1. Untargeted Microbial Metabolomics Assays

Frozen samples stored at −80 °C were thawed on ice and homogenized with a grinder at 30 Hz for 20 s. Briefly, 40 mg of sample was added to 400 μL of internal standard-containing methanol/water solution (4:1, *v*/*v*) and vortexed for 3 min. The sample was cycled three times by sequential freezing in liquid nitrogen for 5 min, cooling on dry ice for 5 min, thawing on ice, and vortexing for 2 min. After pretreatment, the sample was centrifuged at 12,000 rpm for 10 min at 4 °C. Then, 300 μL of the collected supernatant was placed at −20 °C for 30 min and centrifuged again at 12,000 rpm for 3 min at 4 °C. Finally, 200 μL of the supernatant was transferred for LC‒MS analysis.

All the samples were analyzed under both positive and negative ion modes using identical elution gradients. Chromatographic separation was performed on a Waters ACQUITY Premier HSS T3 column (1.8 µm, 2.1 mm × 100 mm; Waters Corporation, Milford, Massachusetts, USA) with 0.1% formic acid in water (solvent A) and 0.1% formic acid in acetonitrile (solvent B). The gradient program was as follows: 5–20% B in 2 min, 20–60% B in 3 min, 60–99% B in 1 min (held for 1.5 min), and then returned to 5% B within 0.1 min and maintained for 2.4 min. The column temperature was 40 °C, the flow rate was 0.4 mL/min, and the injection volume was 3 μL.

The raw mass spectrometry data were converted to mzML format using ProteoWizard (version 3.0.7414). Peak picking, alignment, and retention time correction were performed using the XCMS package (version 4.10.1). Prior to downstream analysis, features with true-absence-of-peak-signal rates exceeding 50% across all experimental groups were discarded. For the retained features, zero-intensity values were handled using a two-tier approach: when a feature exhibited zero intensity in more than 50% of samples, the missing values were replaced with one-fifth of the minimum detected intensity; otherwise, the k-nearest neighbor (KNN) method was applied for imputation. Support vector regression (SVR) was used to correct peak intensities. Metabolite annotation of the filtered and normalized features was achieved by matching MS/MS spectra against an in-house spectral library (MetWare Database), integrated public databases (Metlin, HMDB, KEGG, MoNA, MassBank, etc.), and an AI-predicted library. Metabolites were categorized according to metabolite annotation confidence levels (1a, 1b, 2, 3, 4). Briefly, Level 1a: Metabolite identification based on high-confidence matching against authentic standards with MS1, RT and MS2 parameters. Level 1b: Metabolite identification based on moderate-confidence matching against authentic standards with MS1, RT and MS2 parameters. Level 2: Metabolite identification based on high-confidence matching of MS1, RT and MS2 parameters (without authentic standards). Level 3: Metabolite identification based on moderate-confidence matching of MS1, RT and MS2 parameters (without authentic standards). Level 4: Metabolite identification based on matching of MS1 and RT parameters only; features at this level were excluded from differential analysis. AI-predicted library matches were used only to support Level 2–4 annotations. Variable Importance in Projection (VIP) scores were generated from Orthogonal Projections to Latent Structures Discriminant Analysis (OPLS-DA) using MetaboAnalystR. The OPLS-DA model was validated by permutation testing (*n* = 200) to ensure robustness. Metabolites whose fold change was greater than 2, whose nominal *p* value was less than 0.05 and whose VIP value was greater than 1 were identified as nominally differential metabolites for exploratory analysis. In addition, FDR-adjusted *p* values (Benjamini–Hochberg) were calculated for all annotated metabolites and are provided in Supplementary File S1.

#### 2.6.2. RNA-Sequencing

Libraries were constructed following the strand-specific library preparation protocol [31]. Briefly, total bacterial RNA was extracted via the phenol‒chloroform method, and RNA integrity and total RNA yield were accurately quantified using an Agilent 2100 Bioanalyzer (Agilent Technologies, Santa Clara, CA, USA). For prokaryotic library construction, ribosomal RNA was first depleted from total RNA, followed by ethanol precipitation. RNA fragmentation was performed, after which random hexamer primers were used to synthesize first-strand cDNA. During second-strand cDNA synthesis, dTTP was substituted with dUTP in the reaction buffer. Subsequent procedures, including end repair, A-tailing, adapter ligation, fragment size selection, and PCR amplification and purification, were carried out to generate strand-specific libraries. The constructed libraries were sequenced on the Illumina X Plus platform with a PE150 sequencing strategy.

Raw reads were initially processed using fastp. Reads containing adapters, poly-N sequences and low-quality reads were discarded to obtain clean reads. The Q20, Q30 and GC contents of the clean data were calculated simultaneously, and all subsequent analyses were performed on these high-quality clean datasets. Filtered clean reads were mapped to the reference genome using Bowtie2, with the maximum mismatch number set to 2 and all other parameters kept as default. The read counts mapped to individual genes were quantified via featureCounts. Raw integer read counts generated by featureCounts were used as input for differential expression analysis using the DESeq2 R package. Gene expression levels were normalized to FPKM values according to gene length and mapped read counts. GO and KEGG enrichment analyses of differentially expressed genes were completed using clusterProfiler.

### 2.7. Fluorescence-Activated Cell Sorting (FACS) Analysis

Flow cytometric sorting was performed on a Beckman CytoFLEX SRT instrument fitted with a 100 μm nozzle, using sterile normal saline as the sheath solution. MG1655 *gadA*-*mcherry gadB*-*gfp* was grown in LBK broth at 37 °C with shaking at 220 rpm overnight. Aliquots of the resulting cultures were then transferred at a 1:100 dilution into fresh liquid LBK medium and incubated at 37 °C and 220 rpm for 12 h (stationary phase). Cells were collected, washed, and resuspended in sterile PBS. Intact bacterial populations were distinguished by gating on forward scatter (FSC) versus side scatter (SSC). An unlabeled MG1655 wild-type strain was used as the fluorescence-negative control to establish the autofluorescence baseline. Gates for GadA/GadB-high and -low subpopulations were defined based on dual fluorescence intensity in the B525 (GFP) and Y610 (mCherry) channel. Approximately 6 × 10^5^ events were collected from each fraction. Sorted cells were not re-analyzed for purity and were directly subjected to acid stress and antibiotic treatment. Data were processed using FlowJo V10 software (Tree Star, Inc., Ashland, OR, USA).

### 2.8. Quantitative Real-Time PCR Assay

The tested strains were grown in LBK broth at 37 °C with shaking at 220 rpm overnight. Aliquots of the resulting cultures were then transferred at a 1:100 dilution into fresh liquid LBK medium and incubated at 37 °C and 220 rpm for 3 h (exponential phase), 12 h (stationary phase) or 24 h (death phase). Bacterial pellets were collected by centrifugation, washed with PBS, and processed directly for total RNA isolation using the Bacteria RNA Extraction Kit (R403, Vazyme, Nanjing, China). Purified RNA served as the template for reverse transcription, which was performed with the ABScript Neo RT Master Mix for qPCR with gDNA Remover (RK20433, Abclonal, Wuhan, China). Quantitative real-time PCR was subsequently carried out using the 2× Universal SYBR Green Fast qPCR Mix (RK21203, Abclonal, Wuhan, China) with primer pairs specific for *gadA* (5′-GCTGTTAACGGATTTCCGC-3′ and 5′-GTCGATCCAGTTTTTATTGATCGAC-3′), *gadB* (5′-AGAAGCAAGTAACGGATTTAAGG-3′ and 5′-GTCGATCCAGTTTTTGTTAATGGAT-3′), *gadC* (5′-CTATCCGTTGGCTATGTTACTG-3′ and 5′-AACCGTCCACTCAATTTCTG-3′) and for 16S rRNA (5′-TAGAATTCCAGGTGTAGCGG-3′ and 5′-GGGTATCTAATCCTGTTTGCTC-3′). Relative mRNA abundance was determined by the 2^−ΔΔCt^ method and normalized to 16S rRNA.

### 2.9. Statistical Analysis

Statistical analysis was performed in GraphPad Prism 9 software for Windows. Significance was ascertained by two-tailed Student’s t test or two-way ANOVA followed by Sidak’s multiple-comparison test. Error bars represent the standard deviations of the mean from at least three independent experiments. A value of *p* < 0.05 was considered significant. “*” indicates significant differences (*, *p* < 0.05; **, *p* < 0.01; ***, *p* < 0.001).

## 3. Results

### 3.1. Single-Cell RNA-Seq Reveals a gadA/gadB-High Subpopulation

Isogenic bacterial populations universally exhibit phenotypic heterogeneity, wherein distinct subpopulations display different transcriptomic profiles and physiological phenotypes. This cell-to-cell variation serves as the fundamental basis for persister cell formation. To investigate cell-to-cell heterogeneity within bacterial populations, our group previously developed RiboD-PETRI. This prokaryotic single-cell transcriptomic technique has successfully revealed cellular heterogeneity in biofilm-associated bacterial populations [11]. In this study, we performed dimensionality reduction and clustering analysis on single-cell RNA-seq libraries generated from exponential-phase *E. coli* MG1655 samples constructed in our previous study [11] (Figure S2A–D). Integrated dimensionality reduction and clustering were performed on the two biological replicates, which demonstrated strong concordance and reproducibility across samples (Figure 1A and Figure S5A). We identified a distinct subpopulation designated Cluster 3, consisting of 689 cells and accounting for 3.04% of all cells. To evaluate the robustness of Cluster 3 identification, we performed sensitivity analyses using alternative clustering resolutions (0.1, 0.25, and 0.5), PC numbers (7 and 10) (Figure S3), and different integration methods (Figure S4). Under all tested conditions, the *gadA*/*gadB*-high Cluster was consistently recovered, confirming that our clustering results are robust against variation in these parameters. Then, UMAP visualizations of total UMI counts revealed no substantial differences across clusters, suggesting that the observed clustering is unlikely to be driven primarily by variation in total UMI counts per cell (Figure S5B). We characterized marker genes for each cluster and found that *gadA* and *gadB* genes serve as the marker genes of Cluster 3 (Figure 1C,D and Figure S5C). We further analyzed the expression distribution of *gadA* and *gadB*. Cells with high expression of *gadA* and *gadB* predominantly resided in Cluster 3, while only a small fraction was detected in the other clusters (Figure 1E,F). *gadA* and *gadB* encode glutamate decarboxylases which contribute to bacterial acid resistance [13]. These data reveal that the acid tolerance-associated genes *gadA* and *gadB* exhibit marked cell-to-cell expression heterogeneity within the isogenic population and that their expression levels are highly correlated.

### 3.2. Cell-to-Cell Heterogeneity of GadA and GadB Expressions

To confirm the heterogeneous expression of *gadA* and *gadB* in the bacterial population, a dual-fluorescence reporter strain was constructed. In this strain, GadA was fused with mCherry, and GadB was fused with GFP. To validate the reporter strain, we compared the acid resistance of the parental and *gadA*-*mcherry gadB*-*gfp* strains at pH 2.5, 3.5, and 4.4, and found no significant difference in survival (Figure S6A). We also quantified *gadA* and *gadB* transcript levels across the exponential, stationary, and death phases, with only small fold changes observed between the two strains (Figure S6D). Therefore, these results indicate that the fluorescent fusions do not impair GadA/GadB function or alter their native transcriptional regulation, supporting the use of fluorescence intensity as a relative indicator of GadA/GadB expression levels. We analyzed the fluorescence intensity of this reporter strain at the exponential phase. The relative fluorescence intensities of GadA-mCherry and GadB-GFP exhibited a broad dynamic range (50–1000 for GadA, 300–1000 for GadB), with a small subset of cells displaying high expression and another minor population showing low expression (Figure 2A,B). This observation was consistent with the Cluster 3 subpopulation featuring elevated *gadA*/*gadB* expression identified via single-cell RNA-seq (Figure 1C–F). By comparison, the protein SppA, which is fused with GFP and has low expression heterogeneity and uniform expression across cells, displayed a much narrower fluorescence distribution in the absence of any rare cells with extremely high or low expression levels (600–1000 for SppA) (Figure S7). Single-cell transcriptomic analysis revealed that both *gadA* and *gadB* were enriched within Cluster 3. This indicated a consistent expression pattern between the two genes. To confirm the consistent expression pattern of these two genes, we performed Pearson correlation analysis using the relative fluorescence intensities of GadA-mCherry and GadB-GFP in individual cells. The correlation coefficient was *r* = 0.6880 (95% CI [0.5913, 0.7652], *p* < 0.0001), indicating a positive correlation between the expression of these two proteins (Figure 3A). This result is consistent with our single-cell transcriptomic findings.

We further analyzed the fluorescence intensity of this dual-fluorescence reporter strain at the stationary phase and also observed expression heterogeneity of the GadA and GadB proteins. The fluorescence distribution covered a broad range (150–1000 for GadA and 150–1000 for GadB), with a substantial proportion of cells residing at the high and low extremes of the distribution (Figure 2C,D). The correlation coefficient for the expression levels of the two proteins was *r* = 0.8921 (95% CI [0.8539, 0.9207], *p* < 0.0001), indicating a strong positive correlation (Figure 3B). For death-phase cells, we observed broad fluorescence distributions (50–1000 for GadA and 100–1000 for GadB) and a correlation coefficient of *r* = 0.5395 (95% CI [0.4043, 0.6516], *p* < 0.0001) for the two protein fluorescence intensities (Figure 2E,F and Figure 3C).

In summary, these data support the existence of cell-to-cell heterogeneity in GadA and GadB protein expression throughout the exponential, stationary, and death phases, with a consistent positive correlation between the two protein expression levels.

### 3.3. Enhanced Persister Formation in gadA/gadB Double Deletion Mutants Under Acid Stress

The heterogeneous expression of certain proteins helps bacteria adapt to diverse environmental stresses [32,33,34], such as increasing tolerance to antibiotics. We hypothesized that heterogeneous GadA/GadB expression is associated with antibiotic tolerance. Because of the strong positive correlation between GadA and GadB protein expression levels (Figure 3), the Δ*gadAB* knockout strain, in which GadA/GadB expression is completely abolished, was used as an extreme model for the naturally occurring GadA/GadB-low subpopulation. To investigate whether heterogeneous GadA/GadB expression is associated with persister cell formation, we quantified the persister ratio of the parental strain and the Δ*gadAB* double-knockout mutant across the exponential, stationary, and death phases. The results showed no significant differences in persister ratio between the parental strain and the Δ*gadAB* double-knockout mutant at the exponential, stationary, and death phases (Figure S8A–C). These findings are inconsistent with previous studies [35].

The GadAB system mediates bacterial acid resistance through the consumption of hydrogen ions via glutamate metabolism. We therefore hypothesized that bacteria lacking functional GadAB (either naturally low-expression heterogeneous subpopulations or gene deletion mutants) sustain more severe cellular damage under acid stress. This damage drives entry into a dormant-like state and consequently enhances tolerance to antibiotics. To assess the impact of *gadA*/*gadB* on acid survival, we measured the survival of the BW25113 parental strain and its Δ*gadAB* mutant in LBKG medium adjusted to different pH values. At pH 2.5 and pH 3.5, the parental strain displayed survival rates of 36.87 ± 9.60% and 125.32 ± 45.41%, respectively, whereas the Δ*gadAB* mutant exhibited markedly lower survival rates of 0.03 ± 0.003% and 3.76 ± 0.61%, respectively (Figure S6B). These results indicate that deletion of *gadAB* compromises the acid resistance of *E. coli*. In contrast, exposure to pH 4.4 for 2 h or 5 h did not reduce the viable counts of either the BW25113 parental strain or the Δ*gadAB* mutant (Figure S6B,C). Therefore, we used LBKG medium at pH 4.4 as the acid-stress condition. Stationary-phase bacterial cells were subjected to 2 h of acid stress treatment before persister fraction quantification. The results revealed that the persister proportion was 10^−4.17±0.22^ for the wild-type strain and 10^−2.61±0.16^ for the Δ*gadAB* strain (Figure 4A). Compared with the parental strain, the Δ*gadAB* double-knockout mutant had the highest persister fraction, which was approximately 36.3-fold greater. To determine whether the difference in persister ratio was attributable to the deletion of *gadA*/*gadB*, we complemented the Δ*gadAB* mutant with the *gadA*/*gadB* genes expressed under their native promoter and measured the persister ratio under acid stress. Complementation with *gadAB* significantly reduced the persister ratio of the mutant, from 10^−3.23±0.10^ to 10^−4.08±0.03^ (Figure 4B). These results indicate that deletion of the *gadAB* genes leads to a significant increase in persister levels under acid-stress conditions.

Because the Δ*gadAB* double mutant cannot fully recapitulate the GadA/GadB-low subpopulation, we used FACS to sort GadA/GadB-high and -low subpopulations from the MG1655 *gadA*-*mcherry gadB*-*gfp* reporter strain and measured their persister levels under acid-stress conditions (Figure 4C). The GadA/GadB-high subpopulation exhibited persister ratios of 10^−2.71±0.03^ and 10^−1.69±0.04^ under ampicillin (Amp) and ciprofloxacin (Cip) treatment, respectively, whereas the GadA/GadB-low subpopulation exhibited persister ratios of 10^−1.84±0.09^ and 10^−0.67±0.10^ under the same conditions (Figure 4D,E). These results demonstrate that, under acid stress, the pre-sorted GadA/GadB-low subpopulation exhibits a higher persister frequency than the GadA/GadB-high subpopulation, demonstrating an association between GadA/GadB expression level and subsequent population-level persister formation.

### 3.4. The ΔgadAB Double-Mutant Exhibits Dormant-like Features Under Acid Stress

Under acid stress conditions, the persister level of the *gadAB* mutant strain was markedly higher than that of the parental strain. We thus proposed the following hypothesis: disruption of *gadAB* impairs bacterial acid resistance, rendering bacteria more prone to developing a dormant-like phenotype under acid stress and consequently increasing persister abundance. To verify this hypothesis, we performed transcriptomic and metabolomic profiling of the *gadAB* double-mutant strain under acid stress. Principal component analysis (PCA) of transcriptomic and metabolomic data revealed a clear global distinction between the parental strain and the *gadAB* double mutant (Figure 5A,B).

Genes exhibiting a fold change greater than 2 and a *p-adj* value less than 0.05 were defined as differentially expressed genes. Volcano plots and heatmaps revealed 225 upregulated genes and 60 downregulated genes in the *gadAB* mutant relative to the parental strain (Figure S9A,B). For the upregulated differentially expressed genes, GO enrichment analysis revealed significant enrichment in biological processes, including the cellular response to stress and response to pH, in the *gadAB* mutant strain. This result is consistent with the *gadAB* mutant exhibiting a stronger acid stress response than the parental strain, as exemplified by enrichment of the aspartate family amino acid catabolic process and oxidoreductase activity (Figure 5C). The aspartate family amino acid catabolic process may be associated with the arginine-dependent acid resistance system [36,37]. Oxidoreductase activity may contribute to scavenging ROS induced by acid stress [38]. In addition, quorum sensing was enriched, which has been reported to participate in persister formation [39]. The polyol metabolic process, glycerol metabolic process, and small molecule catabolic process were enriched (Figure 5C). These results indicated that the *gadAB* mutant exhibits profiles consistent with metabolic reprogramming, with molecular signatures consistent with a shift away from active growth toward a dormant-like state under acid stress [40,41]. KEGG enrichment analysis revealed significant enrichment in the degradation of lysine, which is a bacterial acid resistance pathway [36,37] (Figure 5C), further supporting the enhanced acid stress response in the *gadAB* mutant. Enrichment pathways for starch and sucrose metabolism, pentose and glucuronate interconversions, microbial metabolism in diverse environments and propanoate metabolism are consistent with carbon metabolic reprogramming in the *gadAB* mutant, with molecular profiles indicative of a shift away from active growth toward a dormant-like state under acid stress. The enrichment of ABC transporters, glycerophospholipid metabolism and glycerolipid metabolism is consistent with membrane remodeling and altered transport activity under acid stress. On the other hand, for the downregulated differentially expressed genes, GO enrichment analysis showed that multiple transmembrane transport processes were enriched (Figure 5C). This result is consistent with reduced transmembrane transport activity under acid stress. KEGG enrichment analysis showed that the aminoacyl-tRNA biosynthesis pathway was enriched (Figure 5C). This result is consistent with downregulation of translation-associated genes, in line with a low-metabolic, dormant-like state [42].

Metabolites exhibiting a fold change greater than 2, a nominal *p* value less than 0.05 and a VIP greater than 1 were defined as nominally differential metabolites for exploratory analysis. Volcano plots and heatmaps revealed 310 nominally upregulated metabolites and 227 nominally downregulated metabolites in the *gadAB* mutant relative to the parental strain (Figure S9C,D). KEGG enrichment analysis was performed using all the nominally differential metabolites. The results revealed significant enrichment in the glycerophospholipid metabolism pathway (Figure 5D), which was also enriched according to the results of the KEGG enrichment analysis of the transcriptomic data. There were 40 nominally upregulated metabolites and 20 nominally differentially downregulated metabolites in the glycerophospholipid metabolism pathway (Figure 5E). The increase in 15 glycerophosphocholine metabolites and 7 diradylglycerol metabolites is consistent with membrane remodeling, which has been associated with increased membrane compactness in previous studies (Figure 5E) [43,44]. These observations are consistent with membrane remodeling and reduced transport activity in the *gadAB* mutant under acid stress, in line with the transcriptomic findings. The predominant downregulation of phosphatidylethanolamine species (5 downregulated and 3 upregulated, Figure 5E) is consistent with suppressed growth and a dormant-like phenotype [45].

Taken together, the results of integrated transcriptome and metabolome profiling revealed molecular signatures consistent with a dormant-like state in the *gadAB* mutant under acid stress, which may contribute to elevated persister abundance.

## 4. Discussion

### 4.1. Heterogeneous Expression of the gadA and gadB Genes

According to the 2019 Global Burden of Disease (GBD) analysis from the Antimicrobial Resistance Collaborators, bacterial infections are the second most common cause of mortality worldwide [46]. Subsequent research published by this research group further projected that antimicrobial resistance (AMR) will be associated with 8.22 million global fatalities in 2050, with 1.91 million of these deaths directly from AMR [47]. Persister cells create favorable conditions for the evolution of antibiotic resistance in bacteria [48]. As persisters constitute a small heterogeneous subpopulation within bacterial populations, conventional bulk population research cannot capture persisters’ characteristics. Single-cell RNA sequencing is a robust technique for studying heterogeneous subpopulations within bacterial populations. In this study, we analyzed exponential-phase cells of *E. coli* MG1655 and identified a heterogeneous subpopulation with high *gadA* and *gadB* expression (Figure 1). We then constructed dual-fluorescent reporter strains to confirm the heterogeneous expression of GadA and GadB proteins within bacterial populations (Figure 2). In an earlier study, researchers utilized a transcriptional reporter with YFP under the control of the *gadB* promoter. Their experimental outcomes corroborate our present results [49]. However, that study quantified promoter activity of *gadB* only, whereas the present study employed dual-fluorescence protein fusions (GadA-mCherry and GadB-GFP) to simultaneously measure both proteins at the single-cell level, revealing a positive correlation between GadA and GadB abundance within individual cells. Transcriptional regulation of *gadA* and *gadB* is highly complex, with expression modulated by multiple regulators, including RpoS, the cAMP-CRP complex, EvgAS, H-NS, GadE, GadW and GadX. These include but are not limited to the following. (1) Under acid stress, the two-component regulatory system EvgAS activates YdeO, which in turn upregulates *gadE* to induce the expression of *gadA*/*gadB*. (2) The global regulator H-NS directly binds to the GAD boxes of *gadE*, *gadX*, *gadA* and *gadB* to repress the transcription of *gadA*/*gadB*. (3) The carbon catabolite global repressor cAMP-CRP complex inhibits the transcription of *rpoS* and decreases GadX levels. CRP is also capable of occupying the GadX-binding sites upstream of the *gadA*/*gadB* promoter to suppress *gadA*/*gadB* transcription. (4) GadW binds to the *gadX* promoter to repress its transcription. It competes with GadX for binding to the GAD boxes of *gadA*/*gadB*. It can also form heterodimers with GadX to counteract its transcriptional activation activity [15,22,23,24,25,26,27,50]. The level of involvement for each regulator varies depending on the growth phase and medium. Given such intricate regulatory networks, minor variations in microenvironmental conditions or the expression noise of relevant regulatory factors may amplify the ultimate differences in *gadA* and *gadB* expression levels. For instance, the global regulator RpoS is markedly heterogeneously expressed across bacterial populations [51,52]. Nevertheless, this model remains a proposed mechanism rather than a directly demonstrated one, as single-cell variation in the individual regulators was not measured in the present study. Directly quantifying the expression heterogeneity of RpoS, GadE, GadX, GadW, H-NS, CRP, and EvgAS at the single-cell level and correlating it with the observed bimodal GadA/GadB expression would represent an important avenue for future investigation.

On the other hand, we found that the expression levels of *gadA* and *gadB* within a single cell were positively correlated (Figure 3). The promoter regions of both *gadA* and *gadB* contain GAD box motifs, and they are regulated by nearly identical mechanisms [15,26]. The positive correlation between GadA and GadB expression in individual cells, combined with heterogeneous expression across the bacterial population, highlights the heterogeneity of glutamate-dependent acid resistance phenotypes in bacteria. In this heterogeneous bacterial population, single cells with high co-expression of *gadA* and *gadB* showed high acid tolerance, whereas cells with low co-expression of both genes are more sensitive to acid stress. These differences in acid resistance capacity led to different bacterial fates under acid stress, which further resulted in varying degrees of antibiotic tolerance.

### 4.2. The ΔgadAB Strain Showed Increased Antibiotic Tolerance Under Acid Stress

Because *gadA* and *gadB* showed heterogeneous expression within bacterial populations and their expression levels are positively correlated within individual cells, we used the Δ*gadAB* double-knockout strain as an extreme model for the naturally occurring GadA/GadB-low subpopulation. We found that there were no significant differences in persister levels among the Δ*gadAB* double-knockout strain and the wild-type strain during the exponential, stationary, and death phases (Figure S8). This observation differs from the findings of a previous study, which reported that the proportion of persister cells was significantly greater in the *gadB* single-knockout strain than in the parental strain during the stationary phase in the absence of acid stress [35]. In contrast, we found no significant difference among strains during the exponential, stationary, or death phase without acid stress, and the increased persister phenotype of the *gadAB* mutant was observed only under acid stress. This discrepancy may reflect differences in experimental conditions, and our results indicate that the role of GadA/GadB in persister formation is acid-stress-dependent rather than constitutive. Moreover, the optimal catalytic pH of GadA and GadB is approximately 4. The two enzymes exhibit potent proton-scavenging capacity solely under intracellular acidification. If the intracellular pH remains neutral, their catalytic activity decreases substantially, and they lose the capacity to mediate glutamate-dependent acid resistance [53,54].

Under acid stress conditions, persister levels of the double mutants of *gadA* and *gadB* were significantly greater than those of the parental strain, with an approximately 36.3-fold increase observed in the double mutant (Figure 4). Because the Δ*gadAB* mutant represents complete loss of GadA/GadB rather than naturally occurring low expression, we additionally sorted GadA/GadB-high and -low subpopulations by FACS. The GadA/GadB-low subpopulation exhibited a higher persister frequency than the high subpopulation under acid stress (Figure 4C–E). We note that this comparison was performed between the upper and lower extremes (top 1% and bottom 1%) of the fluorescence distribution. A continuous dose–response relationship would require sorting multiple intermediate bins and represents a direction for future work. We further performed transcriptomic and metabolomic analyses, which showed that under acid stress, the *gadAB* mutant exhibits molecular profiles consistent with a dormant-like state. A limitation of this study is that the bulk RNA-seq and metabolomic analyses were conducted only under acid-stress conditions. Consequently, the design cannot fully distinguish baseline differences between WT and Δ*gadAB* from acid-induced changes. A full experimental design incorporating a no-acid control would be required to formally disentangle these effects and represents an important direction for future work. Furthermore, we acknowledge that direct functional assays, including measurements of growth, metabolic activity, membrane permeability, protein synthesis, and single-cell viability, are absent in the present study for validating these multi-omics-derived observations.

Our findings reveal heterogeneous expression of *gadA* and *gadB* within the bacterial population. We speculate that subpopulations with high *gadA* and *gadB* expression may be better equipped to tolerate the extremely acidic niche in the host gastrointestinal tract. In contrast, subpopulations with low-GadA/GadB expression could potentially enter a dormant-like state under acid stress. If such heterogeneity occurs in vivo, these GadAB-low subpopulations might exhibit enhanced survival upon antibiotic exposure in the acidic gastrointestinal tract. We hypothesize that this could represent a “bet-hedging” strategy for bacteria [55,56]. We acknowledge that these extrapolations are based on in vitro experiments using laboratory *E. coli* K-12 strains and remain to be validated in vivo.

At the mechanistic level, this work demonstrates that naturally occurring heterogeneity in a core acid-resistance system is associated with persister formation under acid stress, providing a conceptual framework for understanding how isogenic bacterial populations generate phenotypic diversity to survive environmental fluctuations. While most persister studies have focused on toxin-antitoxin systems and metabolic dormancy, our findings highlight acid-resistance heterogeneity as an additional, underexplored contributor to persister formation under environmentally relevant conditions. Future studies using in vivo infection models will be needed to determine whether this mechanism contributes to antibiotic treatment failure in the acidic gastrointestinal niche.

## 5. Conclusions

The expression of *gadA* and *gadB* is heterogeneous within an *E. coli* population. Furthermore, the expression levels of *gadA* are positively correlated with those of *gadB* in single cells.

The GadA/GadB-low subpopulation exhibits enhanced tolerance to antibiotics under acidic conditions.

## Figures and Tables

**Figure 1:**
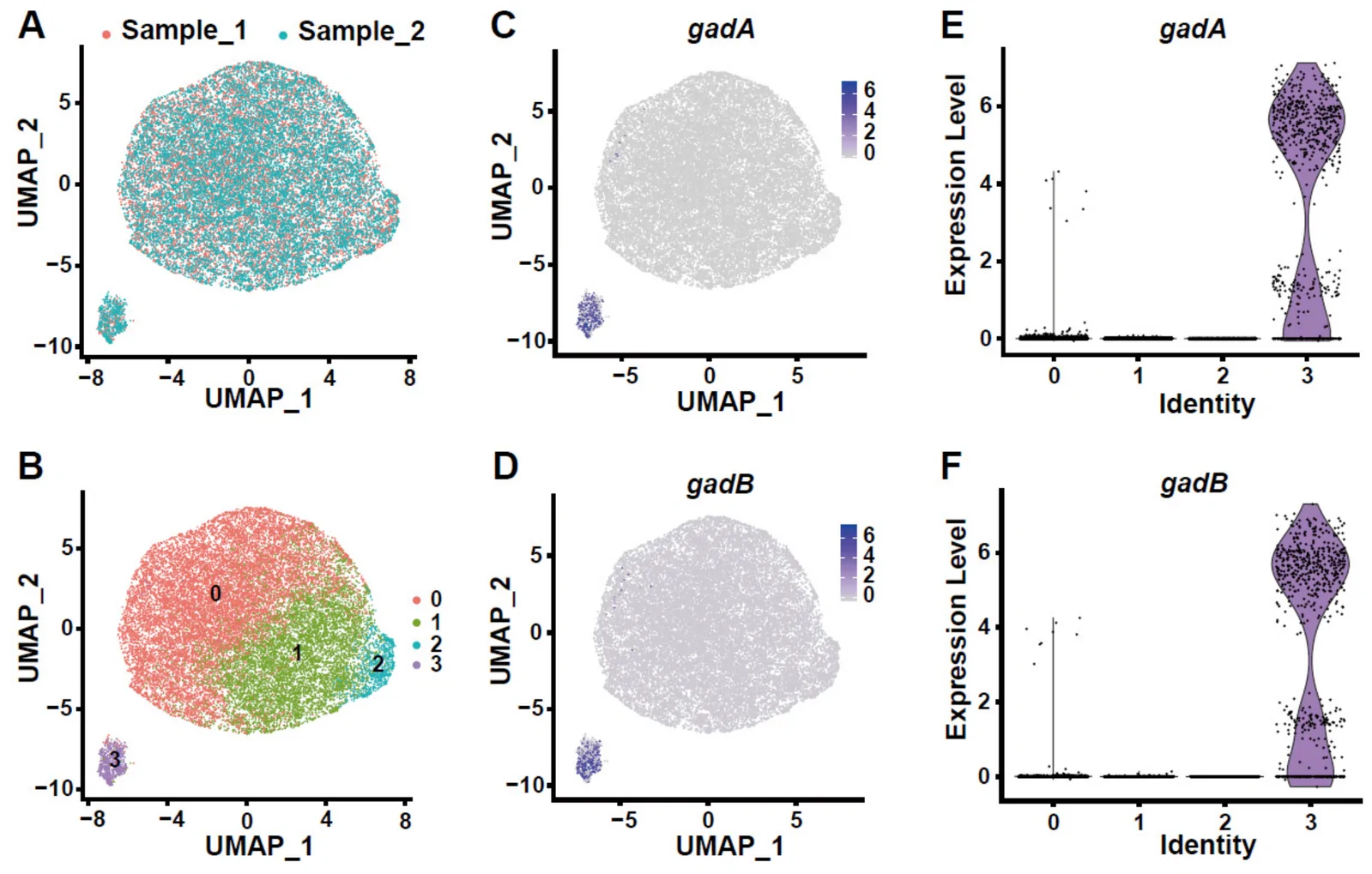
Single-cell transcriptomic analysis of exponential-phase *E. coli* MG1655. Two biological replicate samples were subjected to single-cell RNA sequencing and merged for dimensionality reduction and clustering. (**A**) Uniform manifold approximation and projection (UMAP) embedding of the merged dataset, with cells colored according to the sample of origin (red, Sample 1; blue, Sample 2). (**B**) UMAP visualization of the merged data colored by cluster assignment derived from unsupervised clustering. (**C**) Feature plot showing the expression of *gadA* projected onto UMAP coordinates. (**D**) Feature plot showing the expression of *gadB* projected onto UMAP coordinates. (**E**) Violin plots depicting the expression distribution of *gadA* across the cell clusters identified in (**B**). (**F**) Violin plots depicting the expression distribution of *gadB* across the cell clusters identified in (**B**).

**Figure 2:**
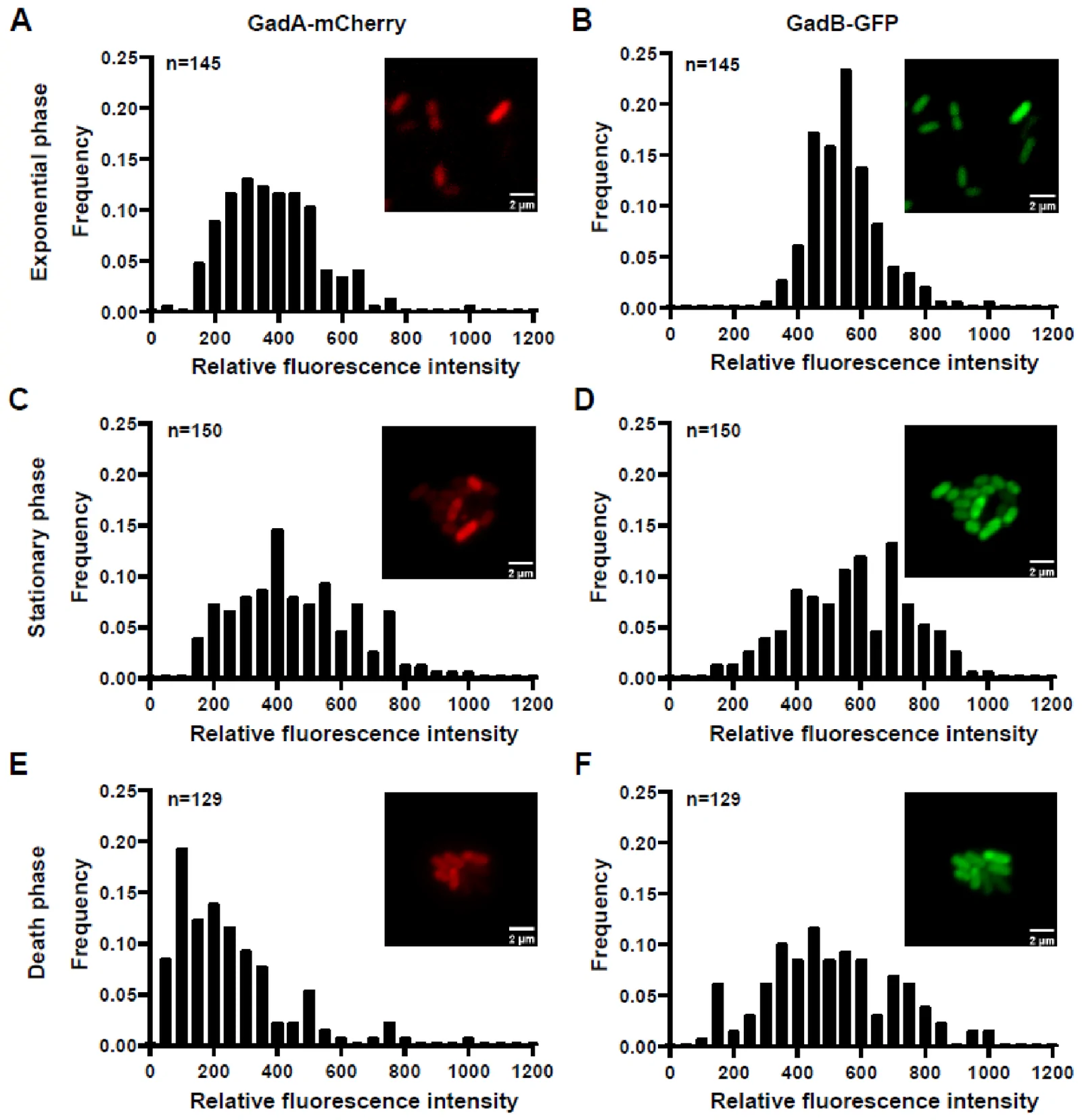
GadA and GadB are heterogeneously expressed at the single-cell level. Cultures of strain MG1655 *gadA*-*mcherry gadB*-*gfp* at the exponential (**A**,**B**), stationary (**C**,**D**) and death phases (**E**,**F**) were imaged by fluorescence microscopy to quantify single-cell mCherry (**A**,**C**,**E**) and GFP (**B**,**D**,**F**) fluorescence intensities as readouts of protein expression. Relative fluorescence intensity was normalized, with the maximum fluorescence signal detected among all cells set to a relative value of 1000. The inset shows representative micrographs of the corresponding samples. The scale bar denotes 2 μm.

**Figure 3:**
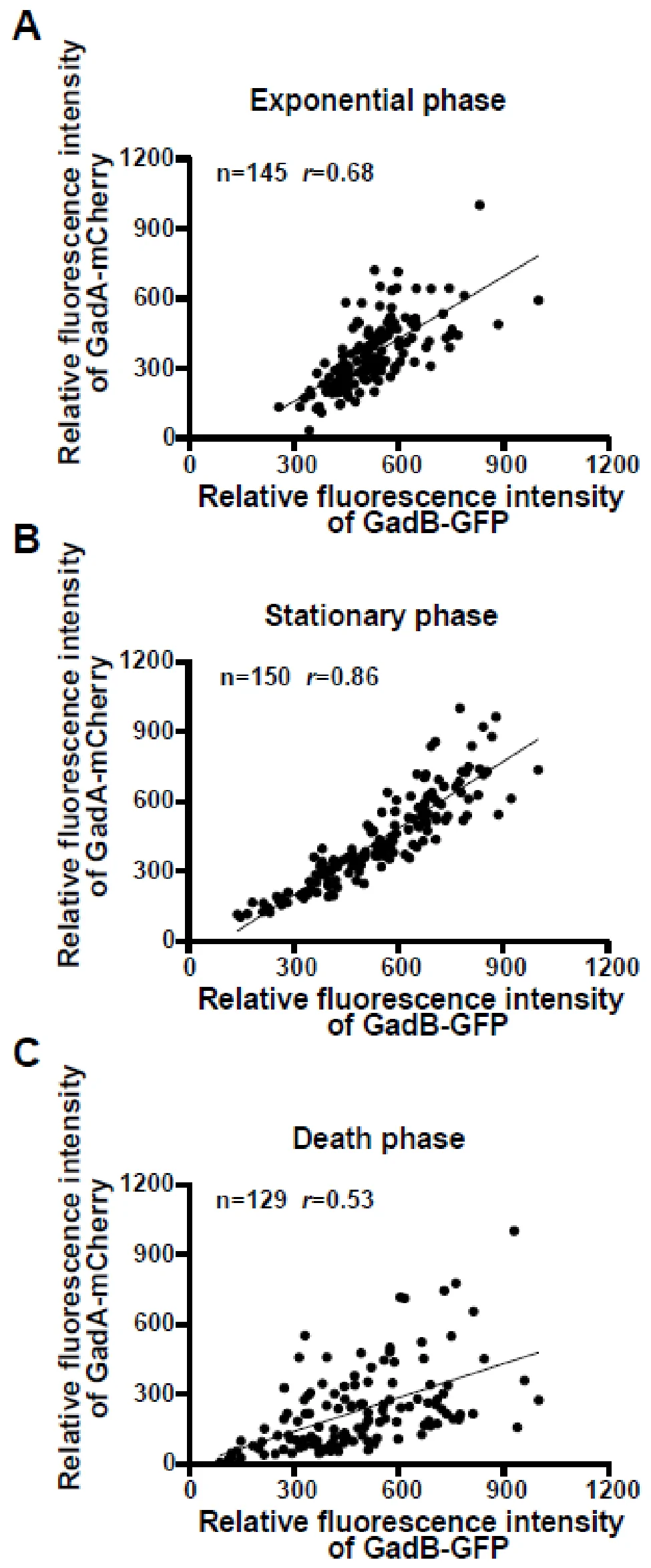
GadA and GadB protein abundances are positively correlated at the single-cell level. Cultures of strain MG1655 gadA-mcherry gadB-gfp at the exponential (**A**), stationary (**B**) and death phases (**C**) were visualized via fluorescence microscopy. Single-cell mCherry and GFP fluorescence intensities were quantified as readouts of GadA and GadB protein expression, respectively. Relative fluorescence intensities were normalized such that the maximum signal across all measured single cells was assigned a relative value of 1000. Each data point in the scatter plot corresponds to an individual bacterium. Pearson correlation analysis was conducted, where r indicates the Pearson correlation coefficient.

**Figure 4:**
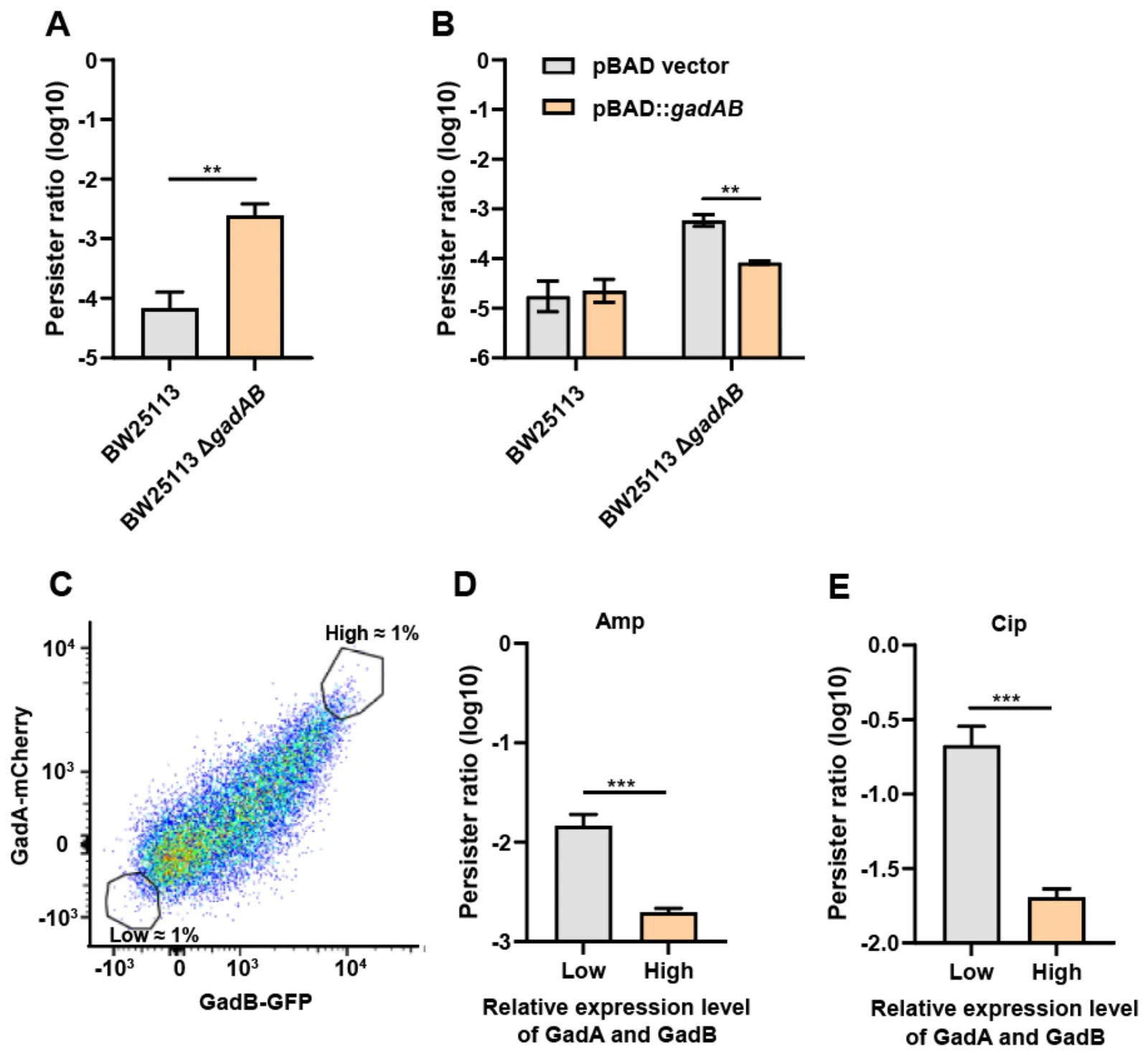
The GadA/GadB-low subpopulation exhibits a higher persister ratio than the GadA/GadB-high subpopulation under acid stress. (**A**) Persister ratios of BW25113 and BW25113 Δ*gadAB*. (**B**) Persister ratios of BW25113 and BW25113 Δ*gadAB* carrying the empty pBAD vector or pBAD::*gadAB*. For A and B, strains were grown to stationary phase, exposed to acid stress in LBKG medium for 2 h, and then subjected to persister quantification (150 μg/mL ampicillin, 3 h at 37 °C with shaking at 220 rpm). (**C**) The MG1655 *gadA*-*mcherry gadB*-*gfp* reporter strain was grown to stationary phase in LBK medium, washed once with PBS, and resuspended in PBS at a 1:1 (*v*/*v*) ratio. The representative FACS plots show the gating of GadA/GadB-high and -low subpopulations (High and Low, ~1% each). The sorted GadA/GadB-high and -low cells were exposed to acid stress in LBKG medium for 2 h, and then subjected to persister assays under ampicillin (Amp) (**D**) or ciprofloxacin (Cip) (**E**). Persister ratios were calculated as the ratio of surviving CFU after antibiotic treatment to the CFU before treatment and are expressed on a log_10_ scale. Error bars represent the standard deviation of three independent biological replicates. Significance was ascertained by two-tailed Student’s t test (**A**,**D**,**E**) or two-way ANOVA followed by Sidak’s multiple-comparison test (**B**). A value of *p* < 0.05 was considered significant. “*” indicates significant differences (**, *p* < 0.01; ***, *p* < 0.001).

**Figure 5:**
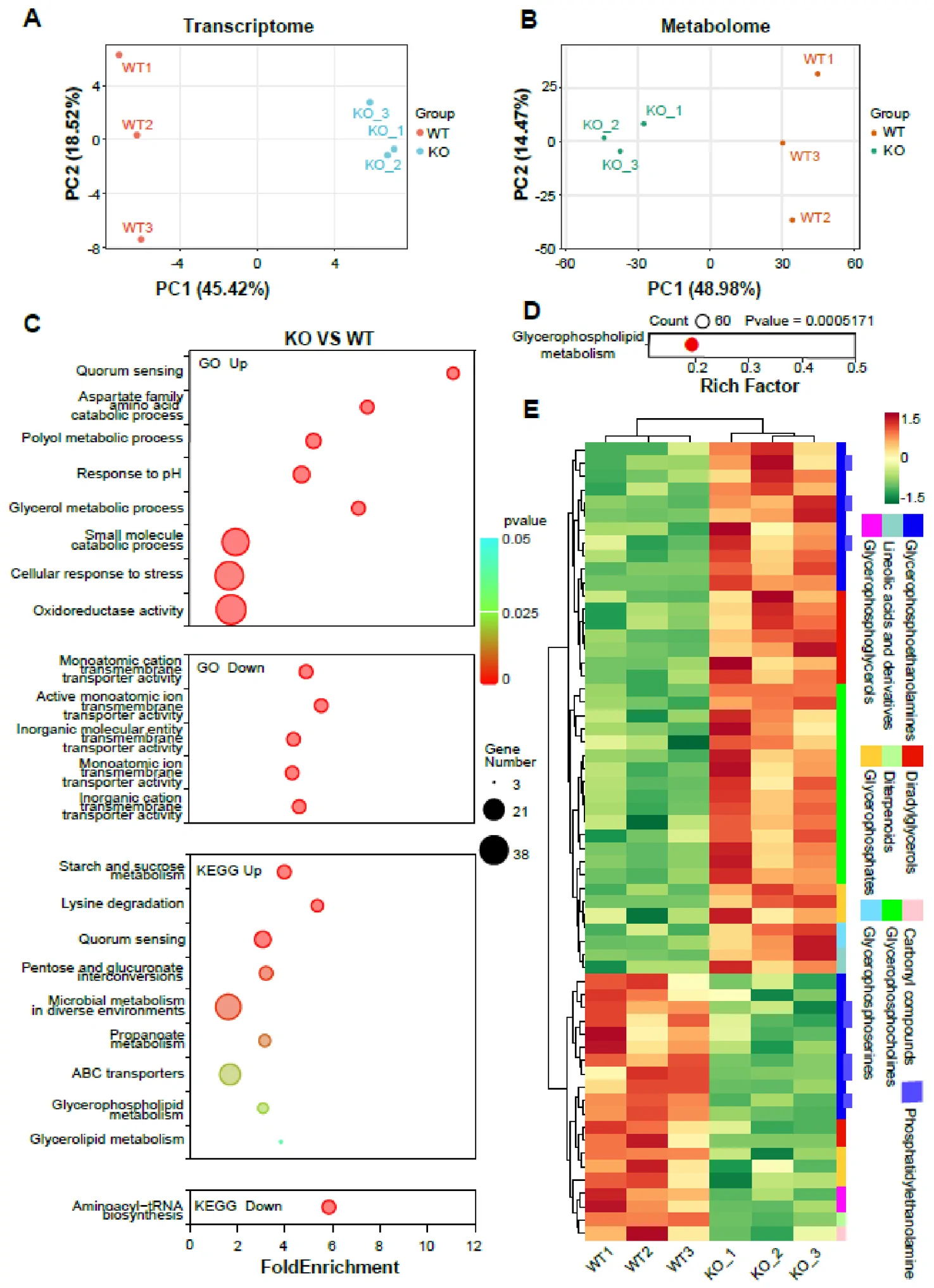
Transcriptomic and metabolomic analyses revealed that the Δ*gadAB* double-mutant strains exhibit a dormant-like state under acid stress. Principal component analysis (PCA) of gene expression profiles (**A**) and metabolite profiles (**B**) in wild-type parental strains and Δ*gadAB* double-mutant strains. (**C**) Genes whose fold change was greater than 2 and whose *p-adj* value was less than 0.05 were selected for GO and KEGG enrichment analysis. (**D**) Metabolites whose fold change was greater than 2, whose nominal *p* value was less than 0.05 and whose VIP value was greater than 1 were screened for KEGG enrichment analysis. (**E**) Heatmap of 60 differentially abundant metabolites involved in the glycerophospholipid metabolism pathway. KO stands for Δ*gadAB*. WT stands for BW25113.

## Data Availability

The intermediate files of single-cell RNA-seq analysis (including gene expression matrices), and raw bulk RNA-seq data generated in this study have been deposited in the NCBI Gene Expression Omnibus (GEO) under accession number GSE337836 (Go to https://www.ncbi.nlm.nih.gov/geo/query/acc.cgi?acc=GSE337836, accessed on 28 September 2026.). The intermediate files and raw metabolomics data generated in this study have been deposited in the Metabolomics Workbench (datatrack_id:7770, study_id:ST005001, Go to https://dev.metabolomicsworkbench.org:22222/data/DRCCMetadata.php?Mode=Study&StudyID=ST005001&Access=TimV7762, accessed on 28 September 2026). All analytical scripts, cell annotation metadata and statistical outputs supporting the figures are available from the corresponding author upon reasonable request.

## Supplementary Materials

The following supporting information can be downloaded at: https://www.mdpi.com/article/10.3390/microorganisms14102180/s1, Figure S1: Time kill curves of *E. coli* BW25113 and MG1655; Figure S2: Dimensionality assessment of the integrated single-cell RNA-seq dataset from exponential-phase *E. coli* MG1655; Figure S3: Sensitivity analysis of clustering parameters; Figure S4: Sensitivity analysis of the integration method; Figure S5: Heatmap of the expressions of the top marker genes across cell clusters in exponential-phase *E. coli* MG1655; Figure S6: Phenotypic characterization of engineered *E. coli* strains: acid-stress survival and *gadA*/*gadB* expression; Figure S7: SppA displays limited expression heterogeneity; Figure S8: Persister ratios of BW25113 and the Δ*gadAB* mutant across growth phases; Figure S9: Volcano plots and heatmaps derived from the transcriptomic and metabolomic data; Table S1: Strains used in this study; File S1: Untargeted metabolomic profiling of the parental (WT) and Δ*gadAB* mutant strains under acid stress.
