# Supplementary material for: Heterogeneously Expressed GadAB Identified by Single-Cell RNA Sequencing Mediates Antibiotic Tolerance Under Acid Stress
Source: Microorganisms. 2026 Sep 29;14(10):2180. doi: 10.3390/microorganisms14102180 (PMC13707565; doi:10.3390/microorganisms14102180)
Supplement: Supplementary file 1 [file microorganisms-14-02180-s001.zip › Figure S1.pdf]

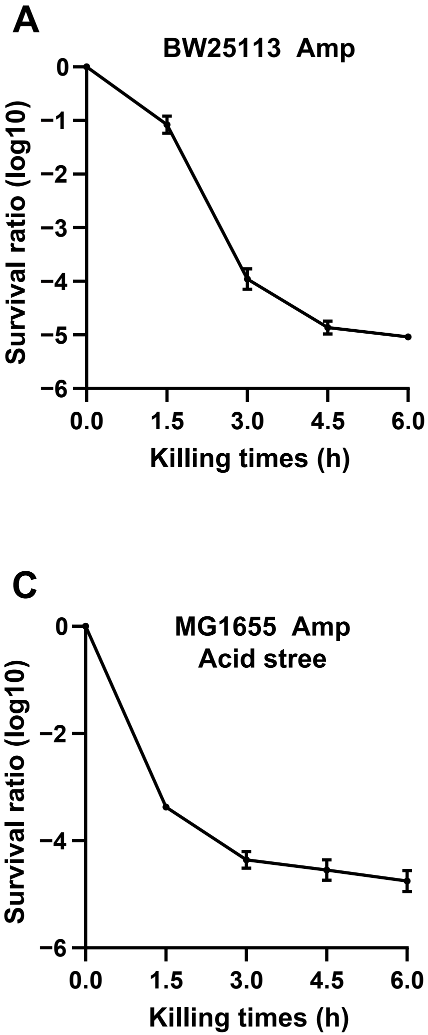

A
BW25113 Amp
Survival ratio (log10)
0
−1
−2
−3
−4
−5
−6
0.0
1.5
3.0
4.5
6.0
Killing times (h)
B
BW25113 Amp
Acid stree
Survival ratio (log10)
0
−1
−2
−3
−4
−5
−6
0.0
1.5
3.0
4.5
6.0
Killing times (h)
C
MG1655 Amp
Acid stree
Survival ratio (log10)
0
−2
−4
−6
0.0
1.5
3.0
4.5
6.0
Killing times (h)
D
MG1655 Cip
Acid stree
Survival ratio (log10)
0
−1
−2
−3
−4
0.0
1.5
3.0
4.5
6.0
Killing times (h)
