# Supplementary material for: Heterogeneously Expressed GadAB Identified by Single-Cell RNA Sequencing Mediates Antibiotic Tolerance Under Acid Stress
Source: Microorganisms. 2026 Sep 29;14(10):2180. doi: 10.3390/microorganisms14102180 (PMC13707565; doi:10.3390/microorganisms14102180)
Supplement: Supplementary file 1 [file microorganisms-14-02180-s001.zip › Figure S2.pdf]

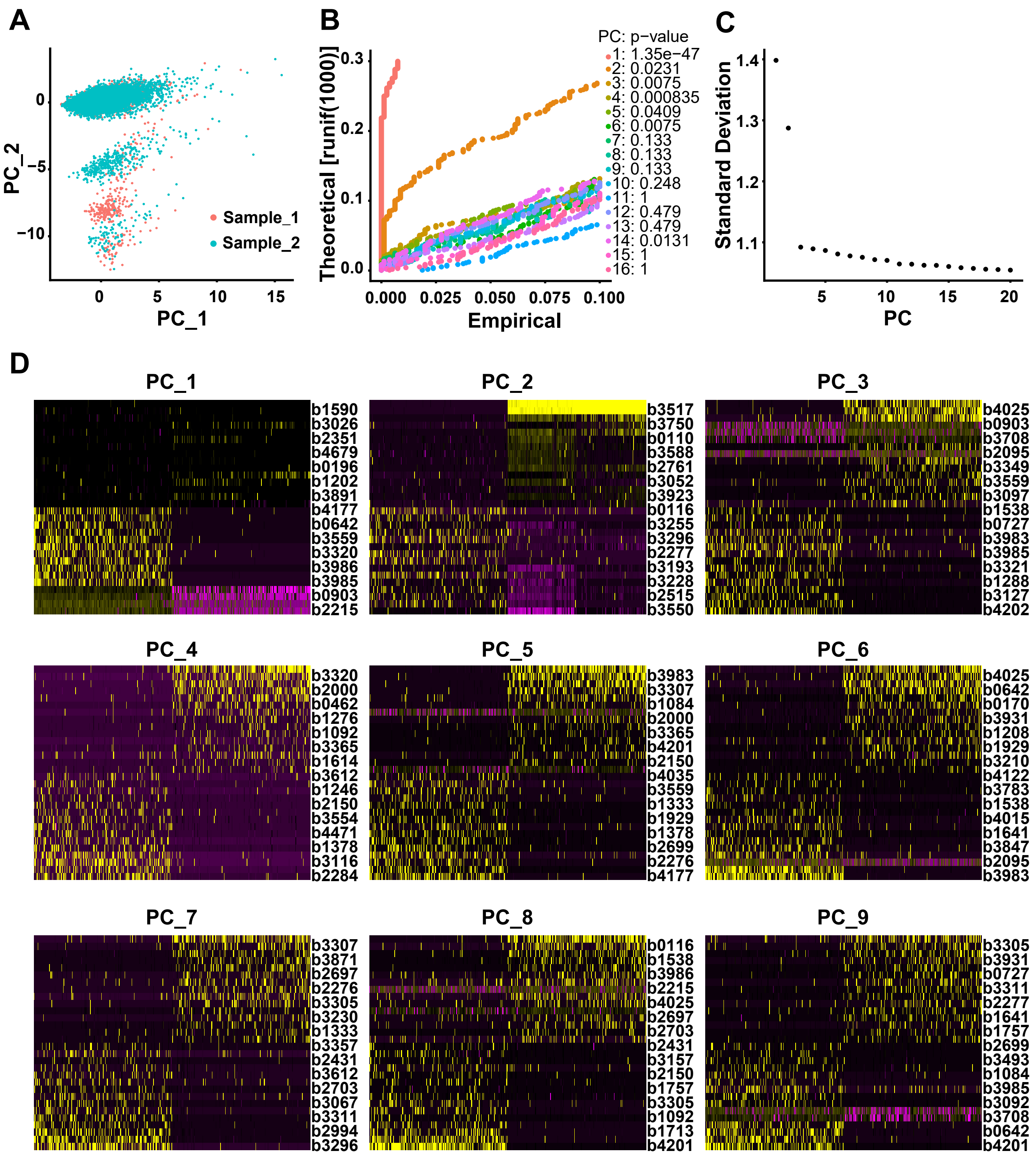
A
PC_2
PC_1
Sample_1
Sample_2
B
Theoretical [runif(1000)]
Empirical
PC: p−value
1: 1.35e−47
2: 0.0231
3: 0.0075
4: 0.000835
5: 0.0409
6: 0.0075
7: 0.133
8: 0.133
9: 0.133
10: 0.248
11: 1
12: 0.479
13: 0.479
14: 0.0131
15: 1
16: 1
C
Standard Deviation
PC
D
PC_1
b1590 b3026 b2351 b4679 b0196 b1202 b3891 b4177 b0642 b3559 b3320 b3986 b3985 b0903 b2215
PC_2
b3517 b3750 b0110 b3588 b2761 b3052 b3923 b0116 b3255 b3296 b2277 b3193 b3228 b2515 b3550
PC_3
b4025 b0903 b3708 b2095 b3349 b3559 b3097 b1538 b0727 b3983 b3985 b3321 b1288 b3127 b4202
PC_4
b3320 b2000 b0462 b1276 b1092 b3365 b1614 b3612 b1246 b2150 b3554 b4471 b1378 b3116 b2284
PC_5
b3983 b3307 b1084 b2000 b3365 b4201 b2150 b4035 b3559 b1333 b1929 b1378 b2699 b2276 b4177
PC_6
b4025 b0642 b0170 b3931 b1208 b1929 b3210 b4122 b3783 b1538 b4015 b1641 b3847 b2095 b3983
PC_7
b3307 b3871 b2697 b2276 b3305 b3230 b1333 b3357 b2431 b3612 b2703 b3067 b3311 b2994 b3296
PC_8
b0116 b1538 b3986 b2215 b4025 b2697 b2703 b2431 b3157 b2150 b1757 b3305 b1092 b1713 b4201
PC_9
b3305 b3931 b0727 b3311 b2277 b1641 b1757 b2699 b3493 b1084 b3985 b3092 b3708 b0642 b4201
