# Supplementary material for: Heterogeneously Expressed GadAB Identified by Single-Cell RNA Sequencing Mediates Antibiotic Tolerance Under Acid Stress
Source: Microorganisms. 2026 Sep 29;14(10):2180. doi: 10.3390/microorganisms14102180 (PMC13707565; doi:10.3390/microorganisms14102180)
Supplement: Supplementary file 1 [file microorganisms-14-02180-s001.zip › Figure S3.pdf]

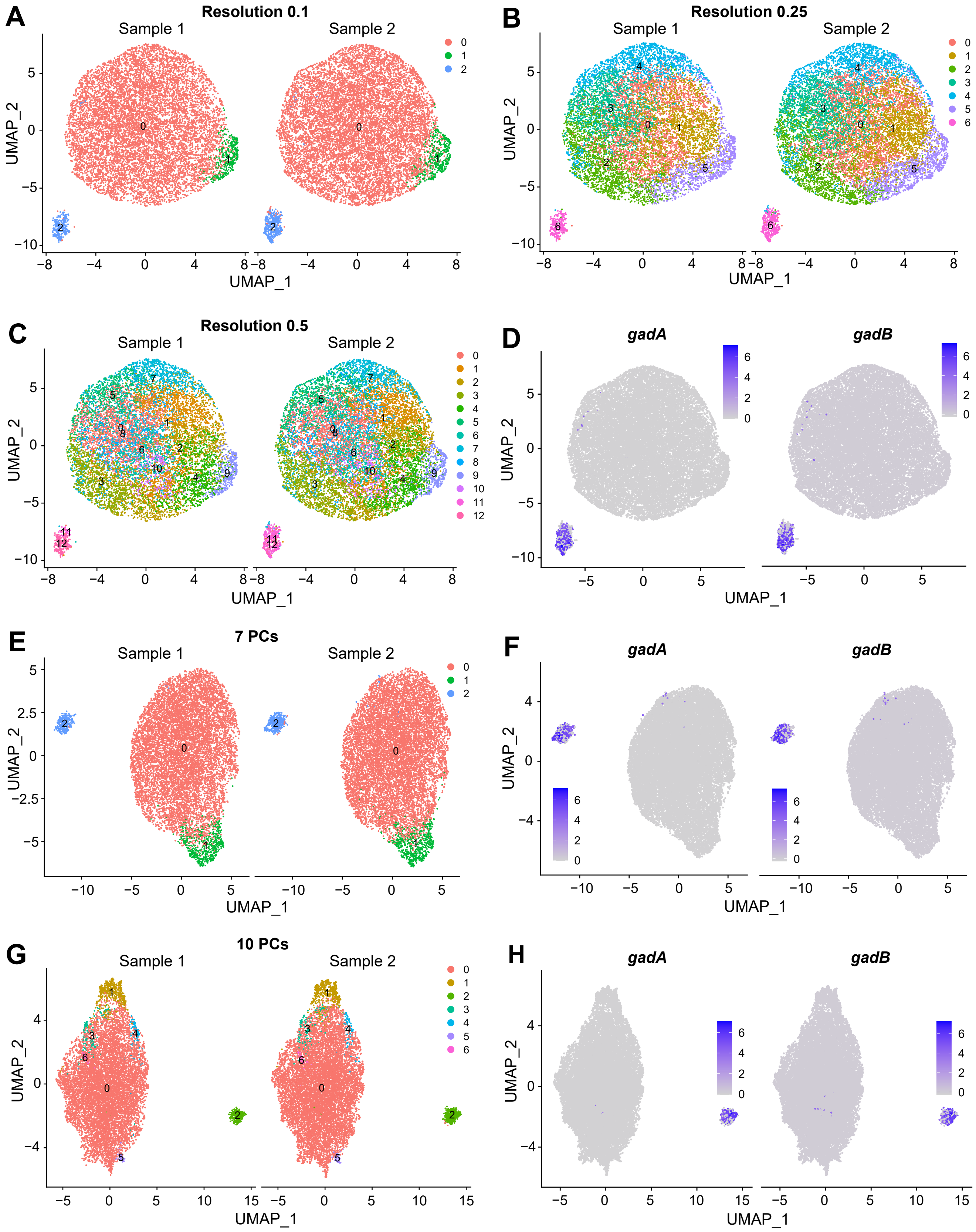
A
Resolution 0.1
Sample 1
Sample 2
B
Resolution 0.25
C
Resolution 0.5
D
gadA
gadB
E
7 PCs
F
G
10 PCs
H
UMAP_1
UMAP_2
