# Supplementary material for: Heterogeneously Expressed GadAB Identified by Single-Cell RNA Sequencing Mediates Antibiotic Tolerance Under Acid Stress
Source: Microorganisms. 2026 Sep 29;14(10):2180. doi: 10.3390/microorganisms14102180 (PMC13707565; doi:10.3390/microorganisms14102180)
Supplement: Supplementary file 1 [file microorganisms-14-02180-s001.zip › Figure S4.pdf]

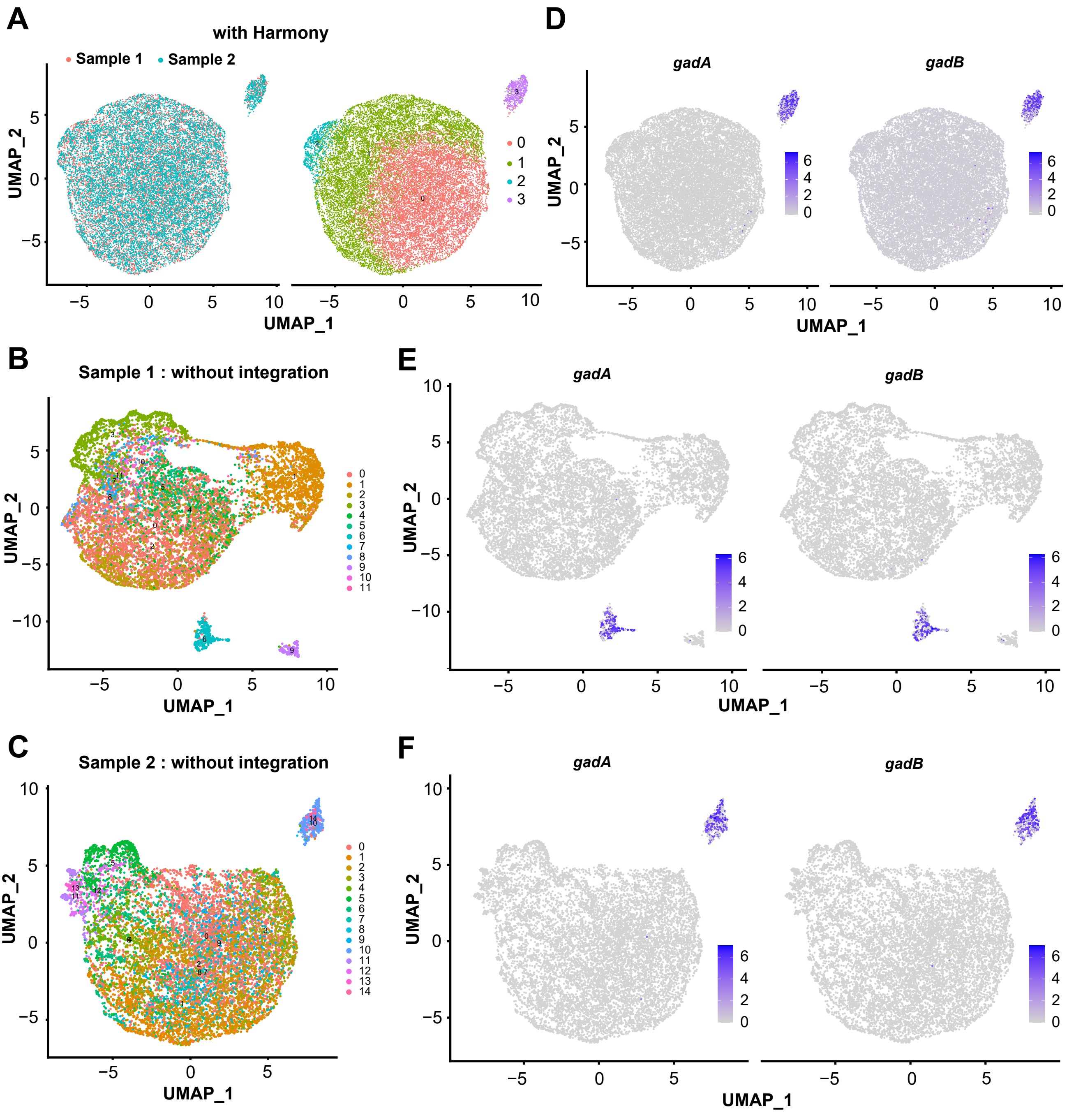

A
with Harmony
Sample 1
Sample 2
UMAP_2
UMAP_1
0
1
2
3
B
Sample 1 : without integration
C
Sample 2 : without integration
D
gadA
gadB
E
gadA
gadB
F
gadA
gadB
