# Supplementary material for: Heterogeneously Expressed GadAB Identified by Single-Cell RNA Sequencing Mediates Antibiotic Tolerance Under Acid Stress
Source: Microorganisms. 2026 Sep 29;14(10):2180. doi: 10.3390/microorganisms14102180 (PMC13707565; doi:10.3390/microorganisms14102180)
Supplement: Supplementary file 1 [file microorganisms-14-02180-s001.zip › Figure S5.pdf]

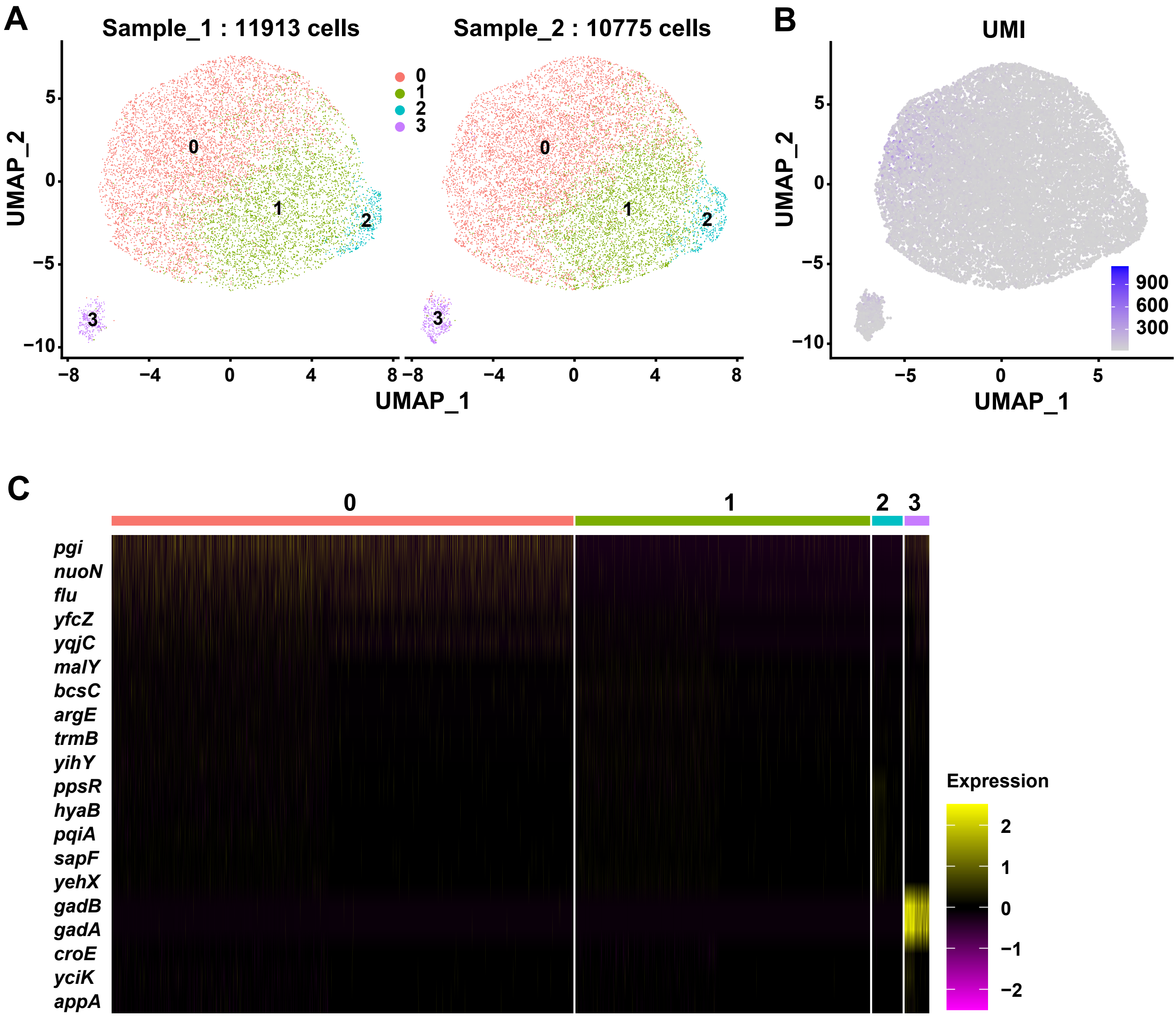
A
Sample_1 : 11913 cells
Sample_2 : 10775 cells
UMAP_2
UMAP_1
0
1
2
3
B
UMI
900
600
300
C
pgi
nuoN
flu
yfcZ
yqjC
malY
bcsC
argE
trmB
yihY
ppsR
hyaB
pqiA
sapF
yehX
gadB
gadA
croE
yciK
appA
Expression
