# Supplementary material for: Heterogeneously Expressed GadAB Identified by Single-Cell RNA Sequencing Mediates Antibiotic Tolerance Under Acid Stress
Source: Microorganisms. 2026 Sep 29;14(10):2180. doi: 10.3390/microorganisms14102180 (PMC13707565; doi:10.3390/microorganisms14102180)
Supplement: Supplementary file 1 [file microorganisms-14-02180-s001.zip › Figure S6.pdf]

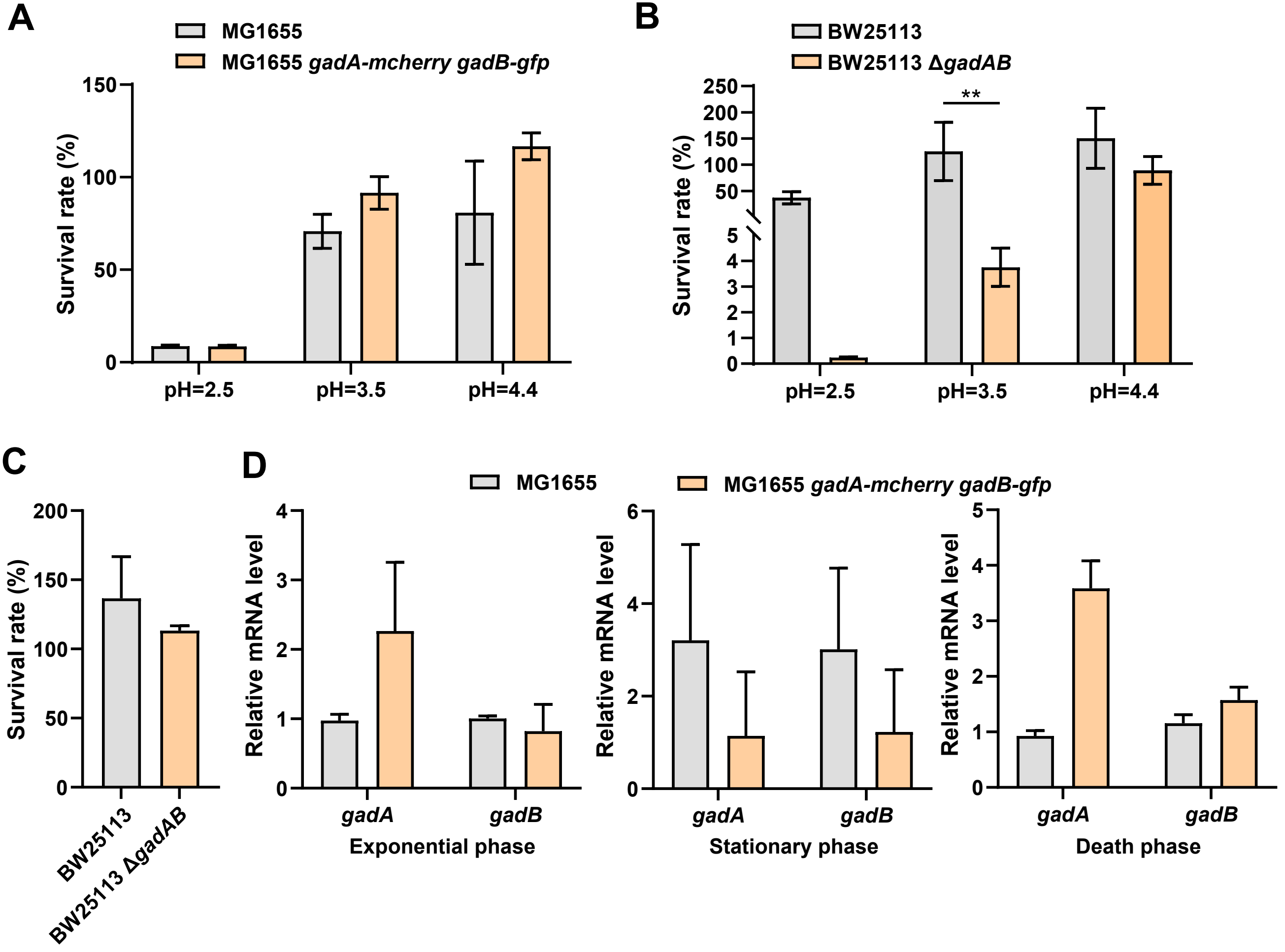
A
MG1655
MG1655 gadA-mcherry gadB-gfp
Survival rate (%)
150
100
50
0
pH=2.5
pH=3.5
pH=4.4
B
BW25113
BW25113 ΔgadAB
Survival rate (%)
**
250
200
150
100
50
5
4
3
2
1
0
pH=2.5
pH=3.5
pH=4.4
C
Survival rate (%)
200
150
100
50
0
BW25113
BW25113 ΔgadAB
D
MG1655
MG1655 gadA-mcherry gadB-gfp
Relative mRNA level
gadA
gadB
Exponential phase
Stationary phase
Death phase
