# Supplementary material for: Heterogeneously Expressed GadAB Identified by Single-Cell RNA Sequencing Mediates Antibiotic Tolerance Under Acid Stress
Source: Microorganisms. 2026 Sep 29;14(10):2180. doi: 10.3390/microorganisms14102180 (PMC13707565; doi:10.3390/microorganisms14102180)
Supplement: Supplementary file 1 [file microorganisms-14-02180-s001.zip › Figure S7.pdf]

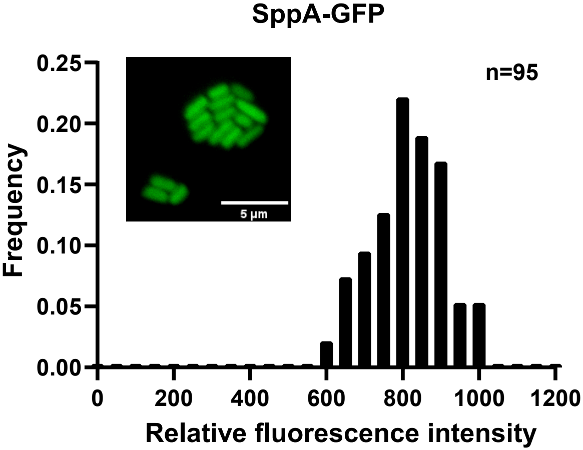
SppA-GFP
n=95
5 µm
Frequency
0.25
0.20
0.15
0.10
0.05
0.00
0
200
400
600
800
1000
1200
Relative fluorescence intensity
