# Supplementary material for: Heterogeneously Expressed GadAB Identified by Single-Cell RNA Sequencing Mediates Antibiotic Tolerance Under Acid Stress
Source: Microorganisms. 2026 Sep 29;14(10):2180. doi: 10.3390/microorganisms14102180 (PMC13707565; doi:10.3390/microorganisms14102180)
Supplement: Supplementary file 1 [file microorganisms-14-02180-s001.zip › Figure S8.pdf]

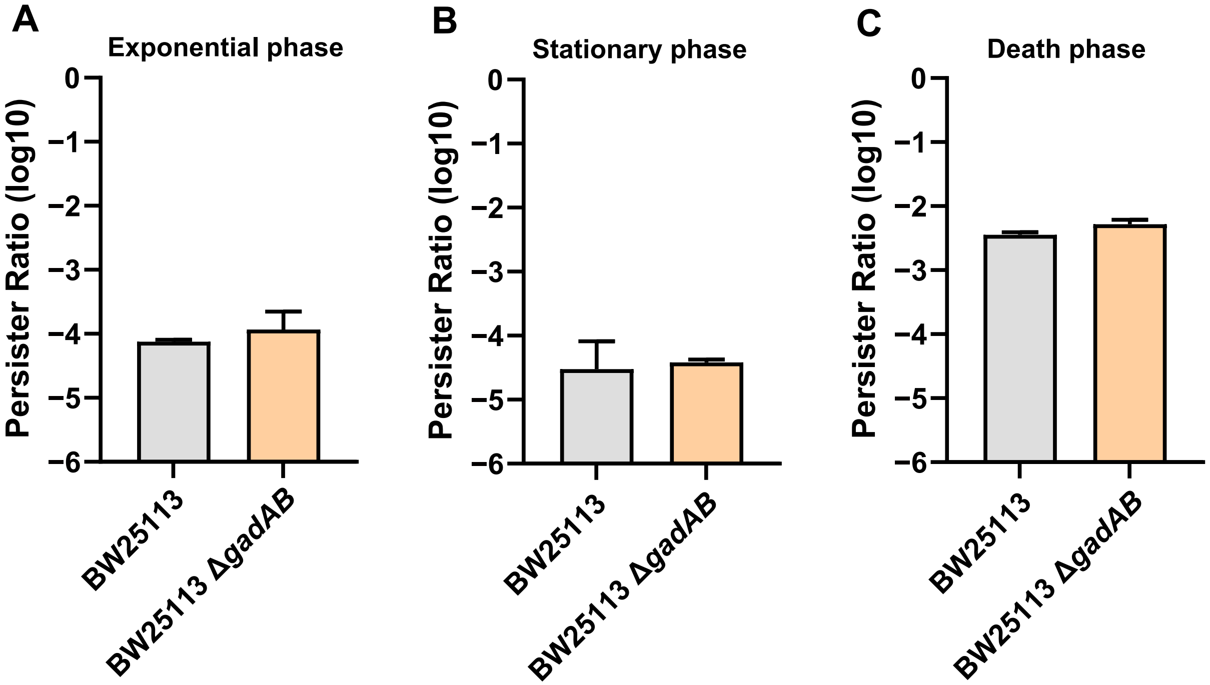
A
Exponential phase
B
Stationary phase
C
Death phase
Persister Ratio (log10)
0
−1
−2
−3
−4
−5
−6
BW25113
BW25113 ΔgadAB
