# Supplementary material for: Heterogeneously Expressed GadAB Identified by Single-Cell RNA Sequencing Mediates Antibiotic Tolerance Under Acid Stress
Source: Microorganisms. 2026 Sep 29;14(10):2180. doi: 10.3390/microorganisms14102180 (PMC13707565; doi:10.3390/microorganisms14102180)
Supplement: Supplementary file 1 [file microorganisms-14-02180-s001.zip › Figure S9.pdf]

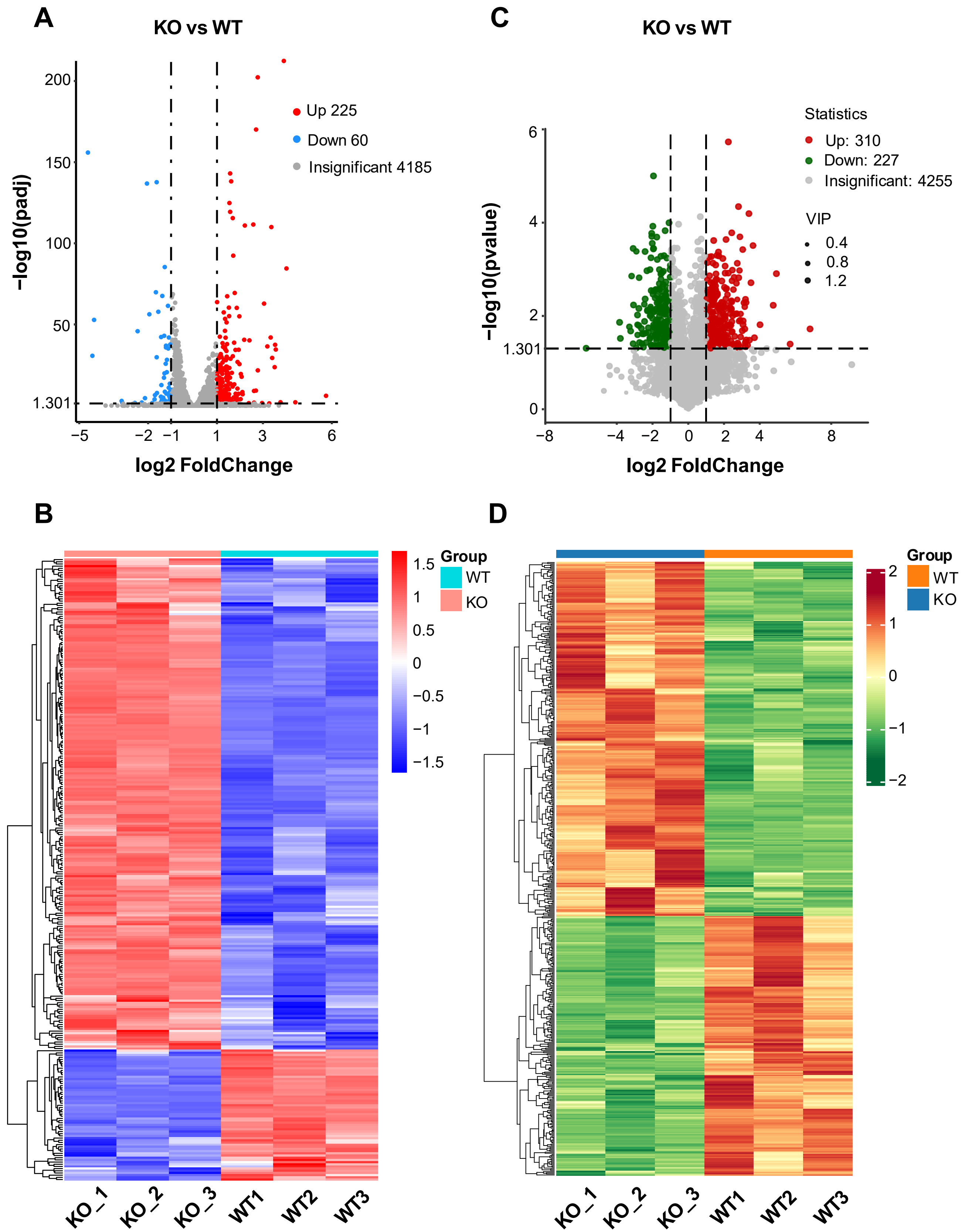
A
KO vs WT
Up 225
Down 60
Insignificant 4185
-log10(padj)
log2 FoldChange
B
Group
WT
KO
KO_1
KO_2
KO_3
WT1
WT2
WT3
C
KO vs WT
Statistics
Up: 310
Down: 227
Insignificant: 4255
VIP
0.4
0.8
1.2
-log10(pvalue)
log2 FoldChange
D
Group
WT
KO
KO_1
KO_2
KO_3
WT1
WT2
WT3
