# Supplementary material for: Heterogeneously Expressed GadAB Identified by Single-Cell RNA Sequencing Mediates Antibiotic Tolerance Under Acid Stress
Source: Microorganisms. 2026 Sep 29;14(10):2180. doi: 10.3390/microorganisms14102180 (PMC13707565; doi:10.3390/microorganisms14102180)
Supplement: Supplementary file 1 [file microorganisms-14-02180-s001.zip › Supplementary Table and Figures.pdf]

Table S1. Strains used in this study

Strain	Description	Source
MG1655	*E. coli* K-12 strain MG1655	Laboratory collection
MG1655 *gadA-mcherry gadB-gfp*	Expression of GadA fused to mCherry and GadB fused to GFP in MG1655	This study
MG1655 *sppA-gfp*	Expression of SppA fused to GFP in MG1655	This study
BW25113	*E. coli* K-12 strain BW25113	Laboratory collection
Δ*gadA*	BW25113 Δ*gadA*::*kanR*	Keio library
Δ*gadB*	BW25113 Δ*gadB*::*kanR*	Keio library
Δ*gadAB*	BW25113 Δ*gadA*::*kanR* Δ*gadB*::*cat*	This study
BW25113 pBAD vector	BW25113 harboring pBAD vector	This study
BW25113 pBAD::*gadAB*	BW25113 harboring pBAD::*gadAB*	This study
Δ*gadAB* pBAD vector	Δ*gadAB* harboring pBAD vector	This study
Δ*gadAB* pBAD::*gadAB*	Δ*gadAB* harboring pBAD::*gadAB*	This study

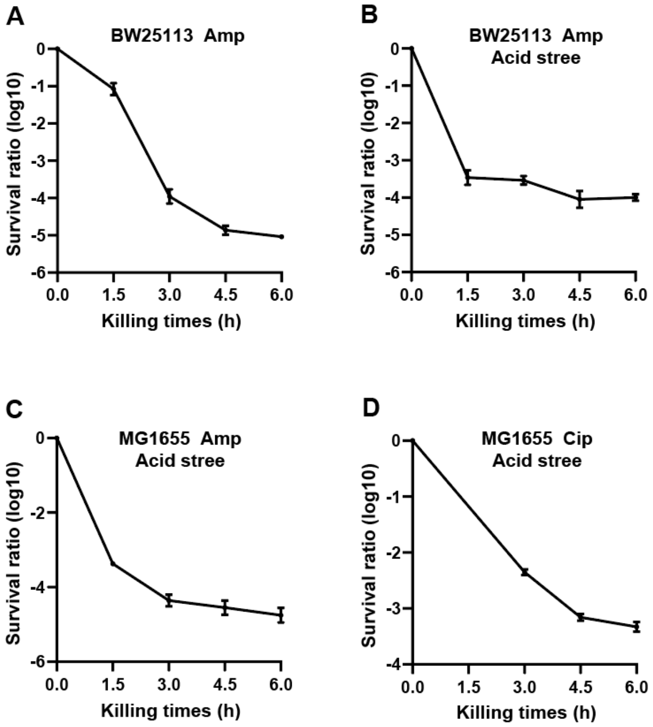

Figure S1. Time-kill curves of *E. coli* BW25113 and MG1655. Both strains were grown to stationary phase in LBK medium. (A) BW25113 cells were diluted 20-fold into fresh LB medium supplemented with 150 μg/mL ampicillin (Amp). (B–D) Cells of BW25113 (B) or MG1655 (C, D) were washed once with PBS, resuspended in LBKG medium at a 1:1 (v/v) ratio, and diluted 1:10 into fresh LBKG medium. After incubation at 37 °C and 220 rpm for 2 h, the cultures were diluted 20-fold into fresh LBKG medium containing 150 μg/mL Amp (B, C) or 3 μg/mL ciprofloxacin (Cip) (D). At the indicated time points, aliquots were collected, washed with PBS, and processed for CFU enumeration. Survival ratios were calculated relative to the initial CFU counts (t = 0) and expressed on a log_{10} scale. Error bars represent the standard deviation of at least three independent experiments.

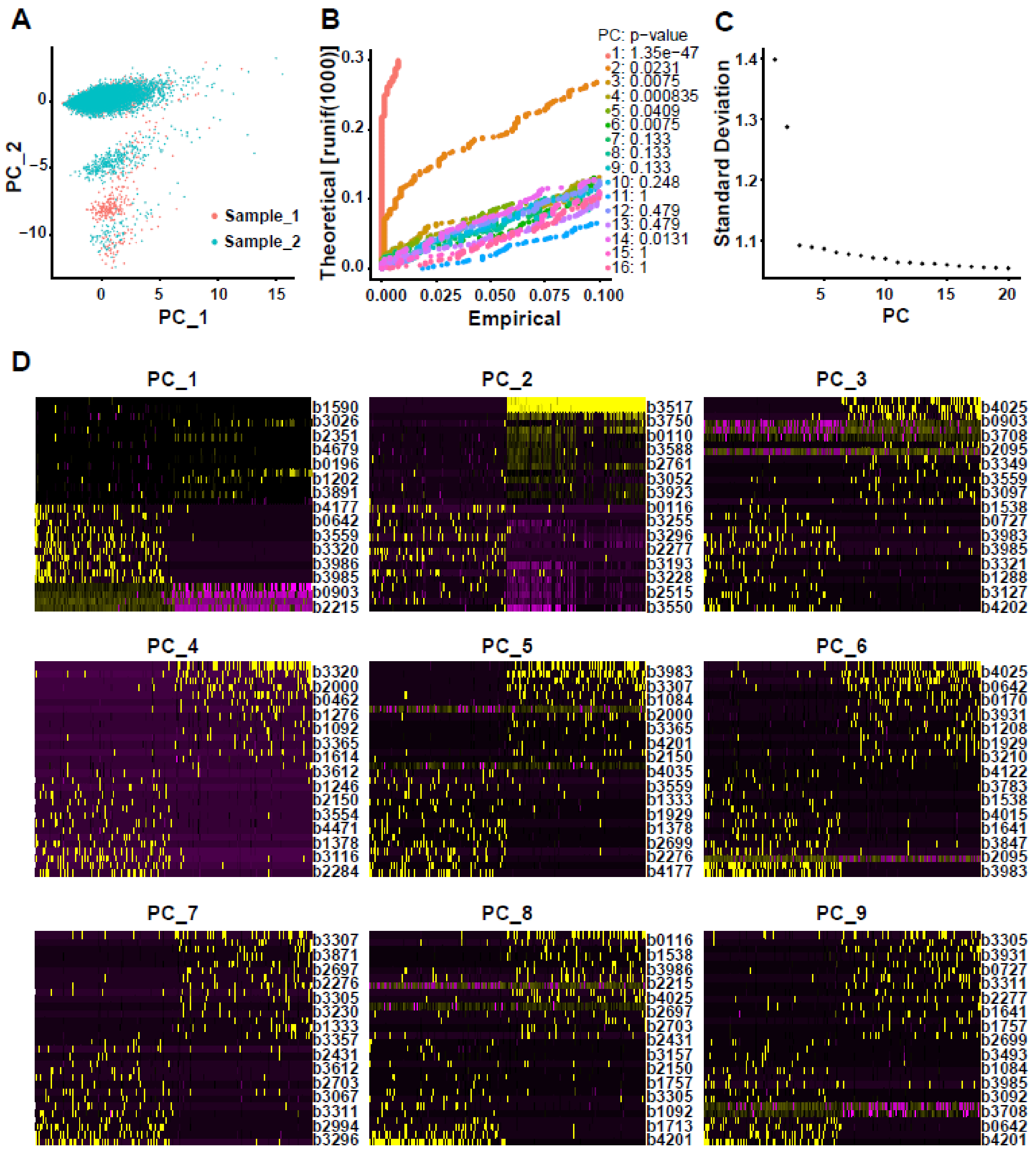

Figure S2. Dimensionality assessment of the integrated single-cell RNA-seq dataset from exponential-phase *E. coli* MG1655. (A) Principal component analysis (PCA) plot of the merged and batch-corrected dataset, with cells colored according to biological replicate (red, Sample 1; blue, Sample 2). (B) JackStraw plot showing the statistical significance of the first 16 principal components (PCs). (C) Elbow plot displaying the standard deviation of the first 20 PCs, used to estimate the number of informative dimensions. (D) Heatmap depicting the expression of the top contributing genes for PCs 1 to 9 across 500 randomly selected cells. Rows represent genes, columns represent cells, and color intensity reflects scaled expression levels.

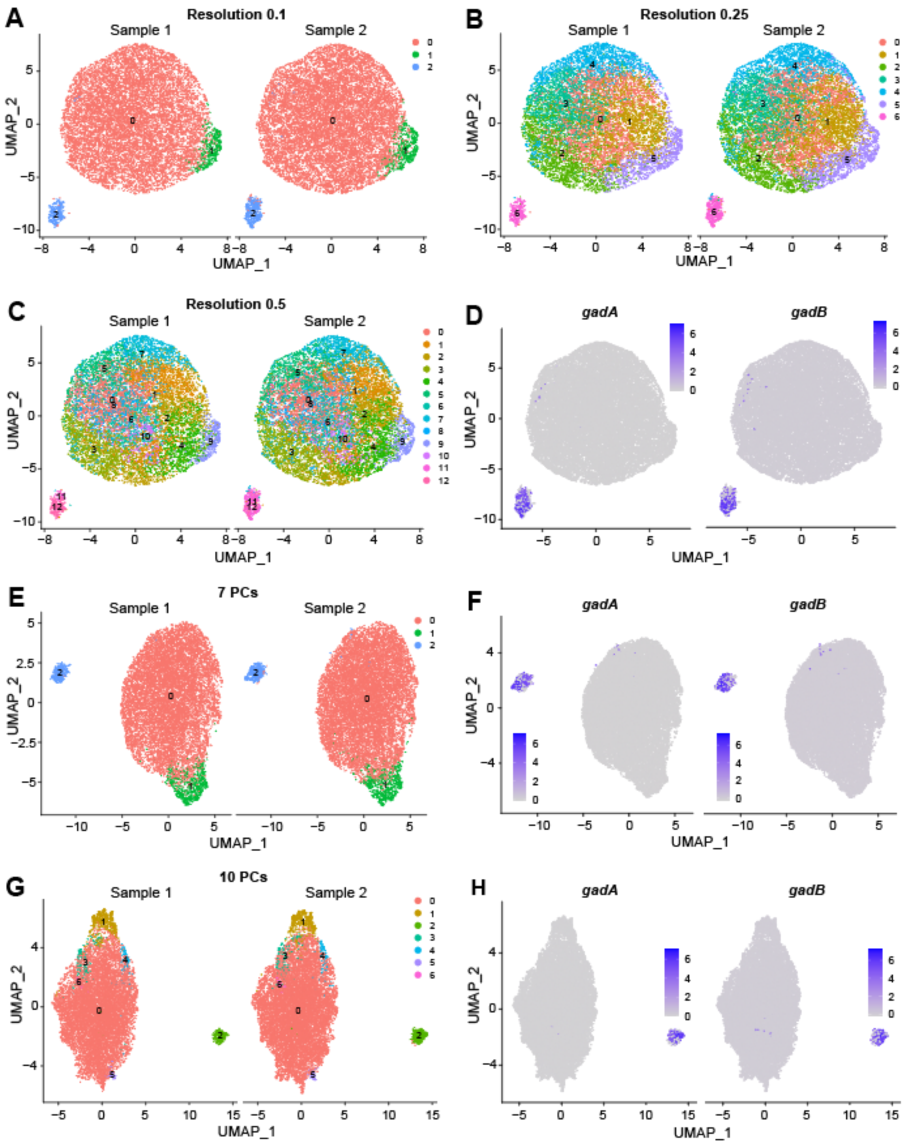

Figure S3. Sensitivity analysis of clustering parameters. UMAP embeddings of the integrated single-cell RNA-seq dataset, colored by cluster identity, are shown for clustering resolutions of 0.1 (A), 0.25 (B), and 0.5 (C), and for 7 (E) or 10 (G) principal components. For each setting, Sample 1 (left) and Sample 2 (right) are displayed as paired plots. (D, F, H) Feature plots showing the expression of *gadA* and *gadB* projected onto the corresponding UMAP coordinates. The *gadA*/*gadB*-high cluster was consistently resolved across all tested parameter combinations.

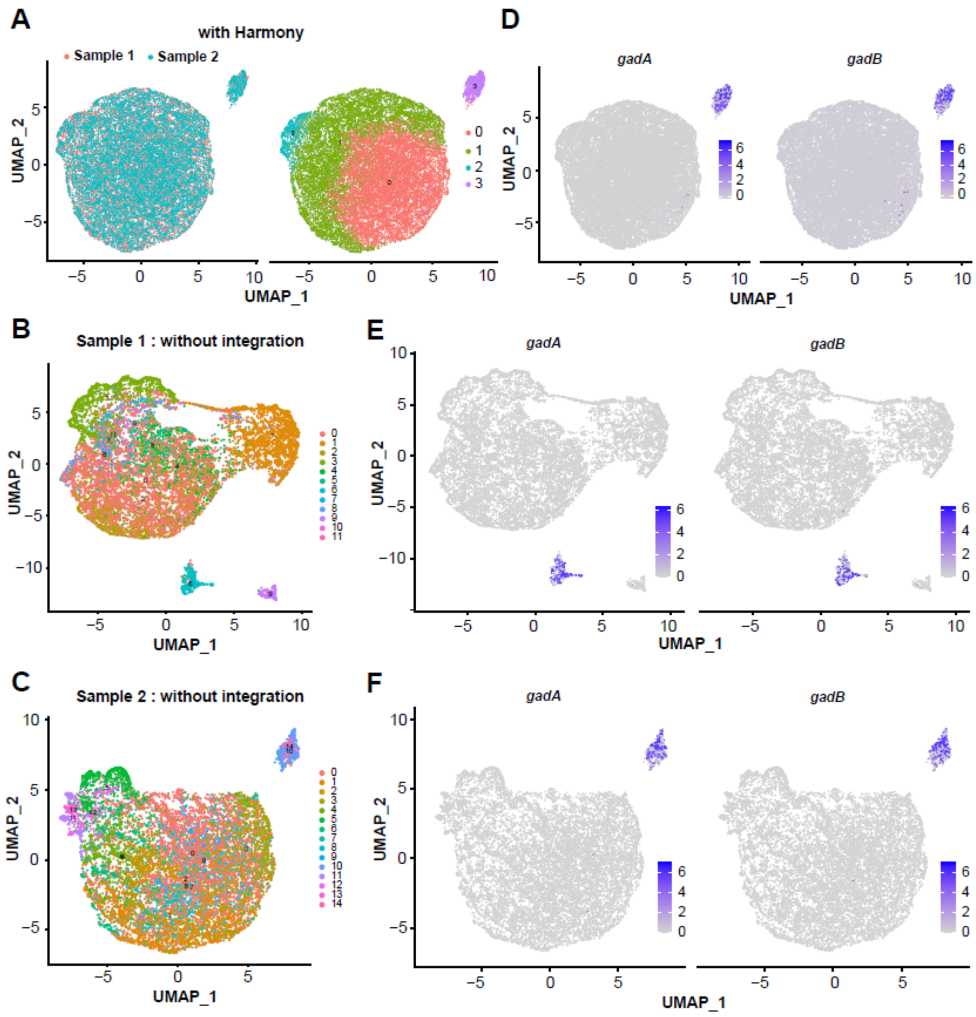

Figure S4. Sensitivity analysis of the integration method. (A) UMAP embeddings of cells from the two samples integrated by CCA with Harmony. Left: cells colored by sample origin, showing batch mixing after CCA with Harmony integration. Right: cells colored by cluster. (B) UMAP embedding of Sample 1 clustered independently without integration, colored by cluster. (C) UMAP embedding of Sample 2 clustered independently without integration, colored by cluster. (D-F) Feature plots showing *gadA* and *gadB* expression on the CCA-with-Harmony embedding (D), in Sample 1 after independent clustering (E), and in Sample 2 after independent clustering (F). In all three workflows, the *gadA*/*gadB*-high cells form a distinct population, confirming that this cluster is not an artifact of the integration method.

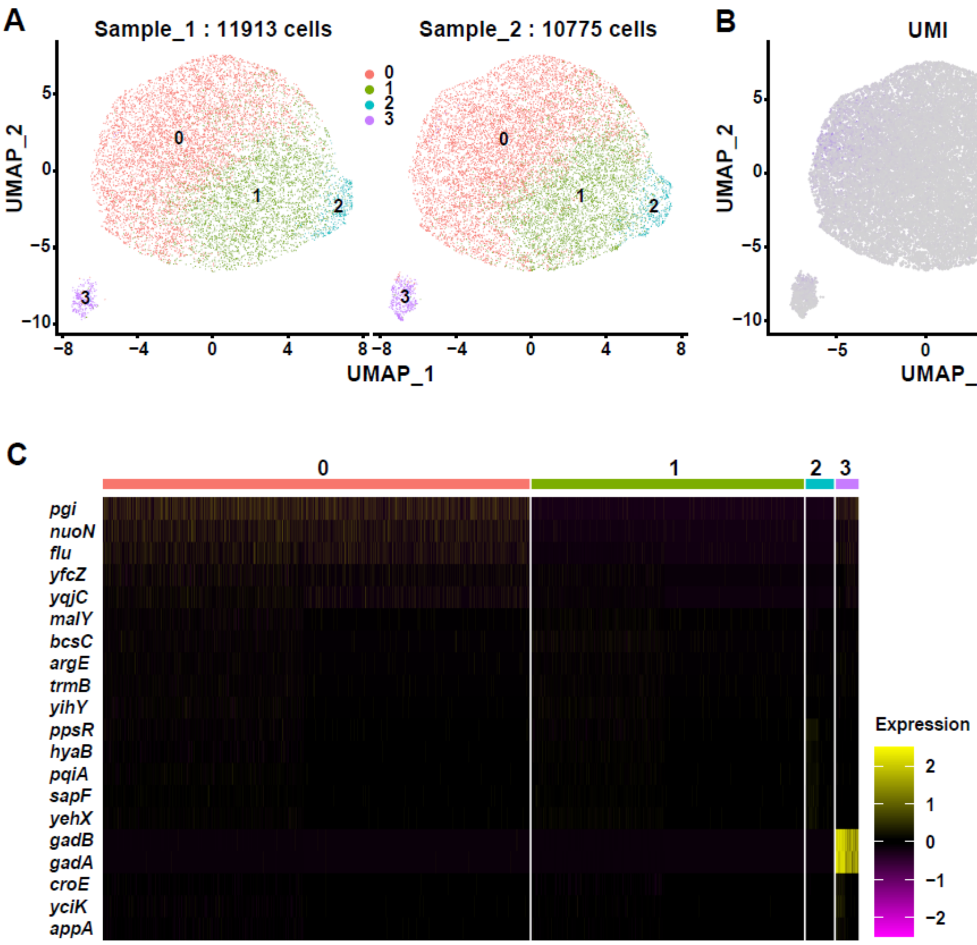

Figure S5. Heatmap of the expressions of the top marker genes across cell clusters in exponential-phase *E. coli* MG1655. (A) Uniform manifold approximation and projection (UMAP) embedding of Sample 1 (left) and Sample 2 (right). (B) Distribution of unique molecular identifiers (UMIs) on the UMAP results for the merged dataset of sample 1 and sample 2 (Figure 1A). (C) The heatmap shows the expression levels of the top 5 marker genes (by average log2-fold change) for each cluster identified in the integrated single-cell RNA-seq dataset. Rows correspond to genes, and columns correspond to individual cells, grouped by cluster. The color scale represents scaled expression levels, with yellow indicating high expression and purple indicating low expression.

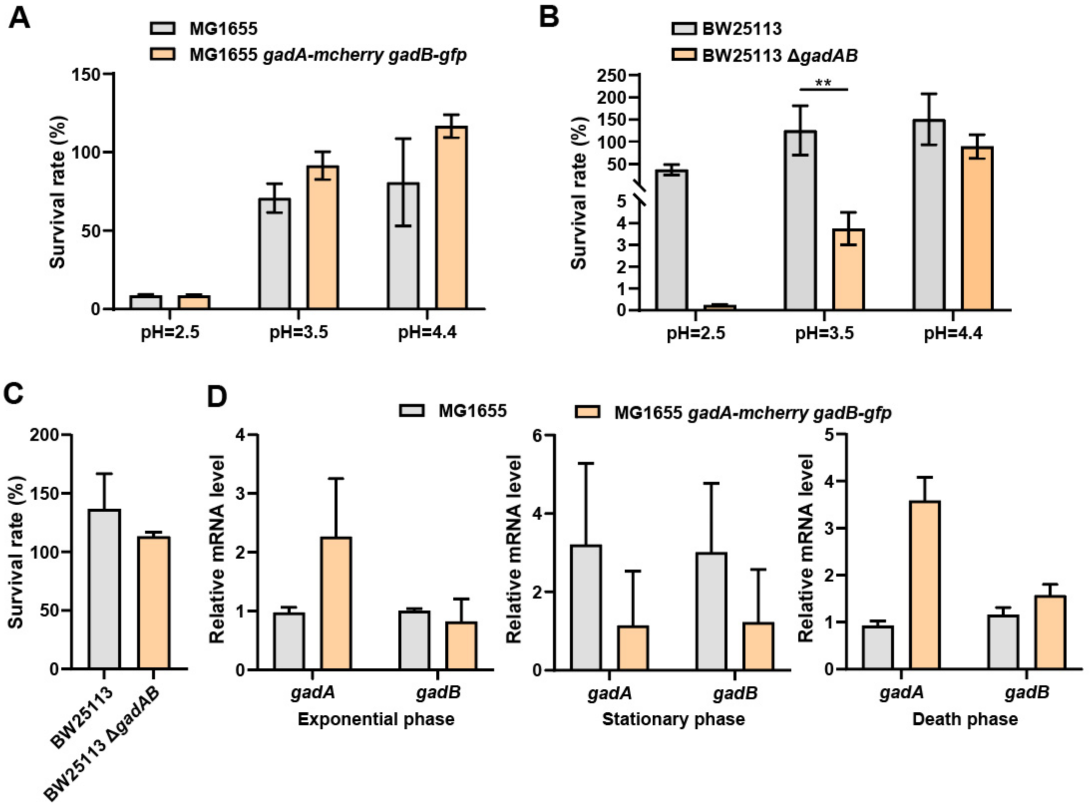

Figure S6. Phenotypic characterization of engineered *E. coli* strains: acid-stress survival and *gadA*/*gadB* expression. (A–C) Acid-stress survival. Strains were grown to stationary phase in LBK medium, washed once with PBS, resuspended at a 1:1 (v/v) ratio in LBKG medium at the indicated pH, and incubated at 37 °C with shaking at 220 rpm for 2 h at pH 2.5, 3.5, or 4.4 (A, B) or for 5 h at pH 4.4 (C). Aliquots were then collected, washed with PBS, and processed for CFU enumeration. Survival ratios were calculated relative to the initial CFU counts before acid stress. (D) Strains were grown in LBK medium to the exponential, stationary, and death phases, after which total RNA was extracted and reverse-transcribed into cDNA for qPCR analysis of *gadA* and *gadB*. Error bars represent the standard deviation of at least three independent experiments. Significance was ascertained by two-way ANOVA followed by Sidak's multiple-comparison test (A-B). A value of $P < 0.05$ was considered significant. "*" indicates significant differences (**, $P < 0.01$).

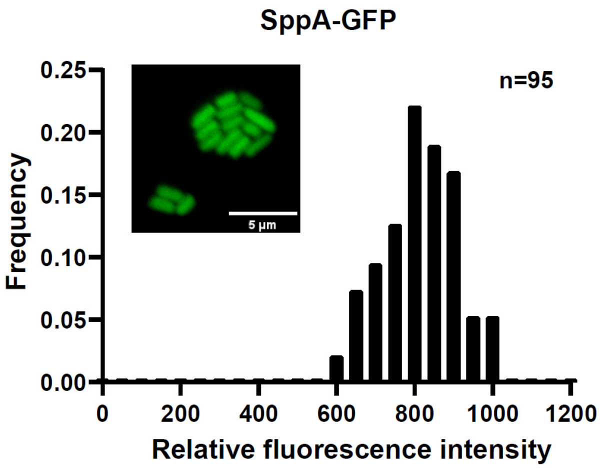

Figure S7. SppA displays limited expression heterogeneity. The cultures of strain MG1655 *sppA-gfp* at the exponential phase were imaged by fluorescence microscopy to quantify single-cell GFP fluorescence intensities as readouts of SppA expression. Relative fluorescence intensity was normalized, with the maximum fluorescence signal detected among all cells set to a relative value of 1000. The inset shows representative micrographs of the corresponding samples. The scale bar denotes 2 μm.

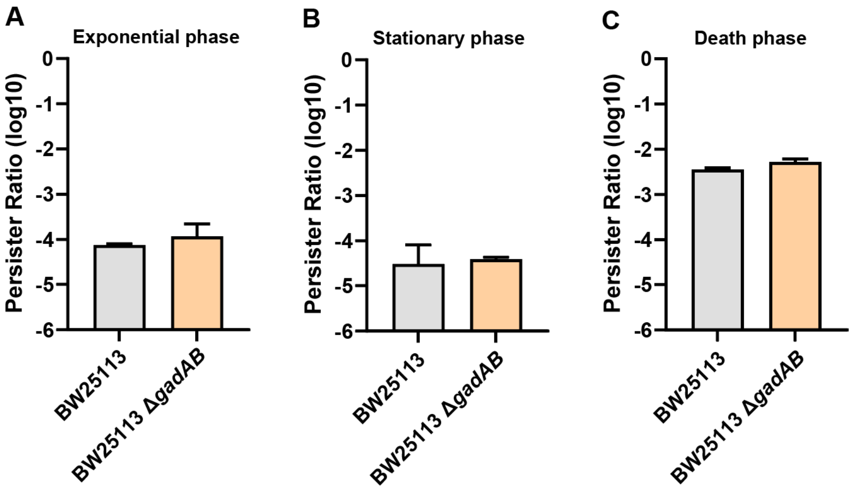

Figure S8. Persister ratios of BW25113 and the Δ*gadAB* mutant across growth phases. BW25113 and BW25113 Δ*gadAB* were grown to the exponential (A), stationary (B), and death (C) phases, and persister assays were performed by exposing the cultures to 150 μg/mL ampicillin for 3 h at 37 °C with shaking at 220 rpm. Persister ratios were calculated as the ratio of surviving CFU after antibiotic treatment to the CFU before treatment and are expressed on a $\log_{10}$ scale. Error bars represent the standard deviation of three independent biological replicates.

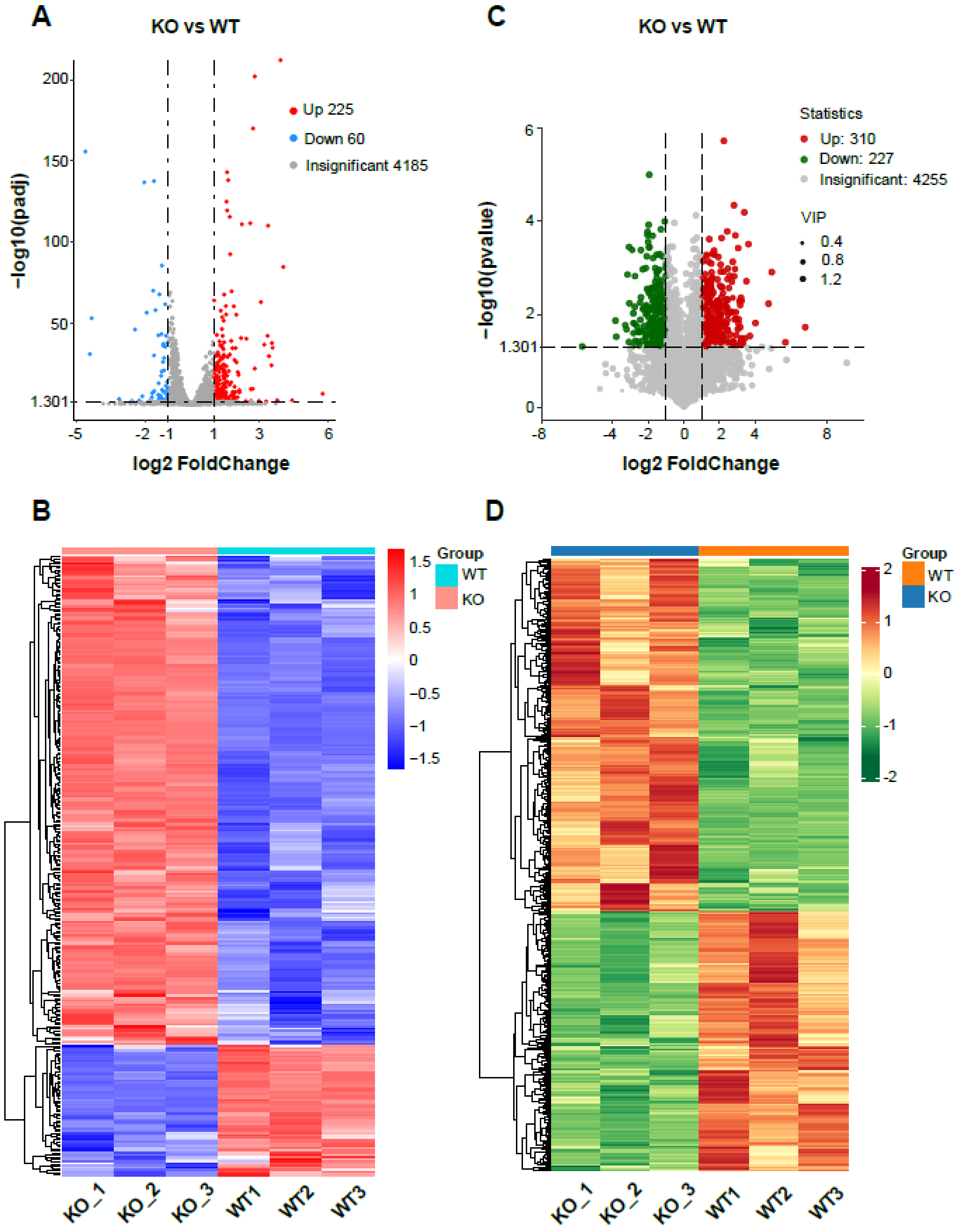

Figure S9. Volcano plots and heatmaps derived from the transcriptomic and metabolomic data. (A) Volcano plots derived from the transcriptomic data. Genes whose fold change was greater than 2 and whose *P-adj* value was less than 0.05 were defined as differentially expressed genes. (B) Heatmap of all differentially expressed genes identified by transcriptome sequencing. (C) Volcano plots derived from the metabolomic data. Metabolites whose fold change was greater than 2, whose nominal *P* value was less than 0.05 and whose VIP value was greater than 1 were defined as nominally differential metabolites. (D) Heatmap of all the differentially accumulated metabolites from the metabolomic analysis. KO stands for Δ*gadAB*. WT stands for BW25113.
